# Microevolution of symbiotic *Bradyrhizobium* populations associated with soybeans in east North America

**DOI:** 10.1002/ece3.404

**Published:** 2012-10-22

**Authors:** Jie Tang, E S P Bromfield, N Rodrigue, S Cloutier, J T Tambong

**Affiliations:** 1 Agriculture and Agri-Food Canada960 Carling Ave, Ottawa, Ontario, Canada, K1A 0C6; 2 Department of Biology, University of OttawaOttawa, Ontario, Canada

**Keywords:** *Bradyrhizobium*, evolution, *Glycine max* (soybean), homologous recombination, inoculation, native legumes

## Abstract

Microevolution and origins of *Bradyrhizobium* populations associated with soybeans at two field sites (A and B, 280 km apart in Canada) with contrasting histories of inoculation was investigated using probabilistic analyses of six core (housekeeping) gene sequences. These analyses supported division of 220 isolates in five lineages corresponding either to *B. japonicum* groups 1 and 1a or to one of three novel lineages within the genus *Bradyrhizobium*. None of the isolates from site A and about 20% from site B (the only site with a recent inoculation history) were attributed to inoculation sources. The data suggest that most isolates were of indigenous origin based on sequence analysis of 148 isolates of soybean-nodulating bacteria from native legumes (*Amphicarpaea bracteata* and *Desmodium canadense*). Isolates from *D. canadense* clustered with *B. japonicum* group 1, whereas those from *A. bracteata* were placed in two novel lineages encountered at soybean field sites. One of these novel lineages predominated at soybean sites and exhibited a significant clonal expansion likely reflecting selection by the plant host. Homologous recombination events detected in the 35 sequence types from soybean sites had an effect on genetic diversification that was approximately equal to mutation. Interlineage transfer of core genes was infrequent and mostly attributable to *gyr*B that had a history of frequent recombination. Symbiotic gene sequences (*nod*C and *nif*H) of isolates from soybean sites and native legumes clustered in two lineages corresponding to *B. japonicum* and *B. elkani* with the inheritance of these genes appearing predominantly by vertical transmission. The data suggest that soybean-nodulating bacteria associated with native legumes represent a novel source of ecologically adapted bacteria for soybean inoculation.

## Introduction

The genus *Bradyrhizobium* includes species of economically important soil bacteria that fix atmospheric nitrogen in symbiotic association with soybeans (*Glycine max*) and thereby minimize the requirement for nitrogen fertilizer inputs in crop production.

In some *Bradyrhizobium* lineages, the accessory genes encoding symbiotic functions (nodulation and nitrogen fixation) reside in a chromosomally located symbiosis island region that has potential for lateral transfer (Kaneko et al. 2002, 2011). The evolutionary histories of genes affecting symbiotic functions in the bradyrhizobia may differ from those of core (housekeeping) genes not directly involved in symbiosis (Stepkowski et al. 2005, 2007; Steenkamp et al. 2008). Therefore, microevolutionary and population genetics studies of bradyrhizobia require separate analysis of symbiotic and core genes.

Soybean is the most important grain legume in the world on a production basis and is a major source of oil and protein. This legume was domesticated in China about 4000 years ago with subsequent cultivation in secondary centers of domestication including India, Thailand, Japan, Korea, and Indonesia (Smartt and Hymowitz 1985). Soybeans were first introduced into the Americas in the late 18th century, but commercial production did not start in the United States and Canada until the 1920s (http://www.soyinfocenter.com/bibliographies.php). Most modern soybeans originate from a narrow genetic base and consequently have limited genetic variability (Delannay et al.1983). Therefore, it is not surprising that only four lineages in the genus *Bradyrhizobium* (*B. japonicum*, *B. elkani*, *B. liaoningense*, and *B. yuanmingense*) have been found to nodulate soybeans under field conditions and all have been encountered in the primary and/or secondary centers of soybean domestication (e.g., Appunu et al. 2008; Vinuesa et al. 2008; Li et al. 2011). As soybean is exotic to North America, soils without a cropping history of this legume are considered to contain few symbiotic bacteria capable of nodulating soybeans (Weaver et al. 1972; Semu et al. 1979). Consequently, soybeans introduced into new environments are inoculated with effective nitrogen-fixing strains of *Bradyrhizobium*. Early work in the United States employing nonmolecular methods such as serotyping suggest that introduced strains often occupy a high proportion of root nodules in the first season, but are progressively replaced in subsequent seasons by heterogeneous symbiotic bacteria resident in soil (reviewed by Streeter 1994). As a result of enrichment by the host plant, populations of bradyrhizobia have become well established in soils of soybean-growing regions in the United States (Weaver et al. 1972).

Diverse native legumes are distributed throughout the soybean growing regions of North America that are known to associate with populations of bradyrhizobia (Sterner and Parker 1999; Parker and Kennedy 2006). For example, *Amphicarpaea bracteata* (Hog Peanut) is a close relative of soybean (Doyle and Doyle 1993; Zhu et al. 1995) and plant infection tests have indicated that *Bradyrhizobium* isolates from this host readily elicit root nodules on soybeans (Marr et al. 1997). Despite these observations, the origins of soybean-nodulating bacteria that frequently outcompete introduced strains in soybean crop ecosystems have yet to be investigated.

Previously, we made a collection of symbiotic bacteria (220 isolates) that were isolated from soybean cultivars inoculated with soil from two field sites (A and B, about 280 km apart in eastern Canada) with contrasting histories of soybean cultivation and *Bradyrhizobium* inoculation. Simultaneously, we assembled a reference collection of *Bradyrhizobium* strains known to have been used in inoculants for soybeans in Canada. During the course of this study, we observed consistent nodulation of soybeans following inoculation with root-zone soils collected from native legumes (*A. bracteata* and *Desmodium canadense*) growing in natural woodland habitats. A further collection (148 isolates) of these soybean-nodulating bacteria was made for comparative analysis.

Our objectives were to infer the microevolutionary histories of bradyrhizobia at soybean field sites A and B as well as the extent to which they originated from inoculation sources or from populations of symbiotic bacteria associated with legumes native to eastern Canada. As recombination is a major evolutionary force that influences the structure of bacterial populations (Fraser et al. 2010), we investigated the effect of homologous recombination on genetic diversification of bacteria from sites A and B.

Genetic characterization of bradyrhizobia was by multilocus sequence typing (MLST) (Maiden 2006) of six core (*atp*D, *gln*II, *rec*A, *gyr*B, *rpo*B, and *dna*K) and two symbiotic (*nod*C and *nif*H) genes. Microevolutionary histories and recombination events were inferred using model-based Bayesian approaches that take into account the effect of recombination (Pritchard et al. 2000; Didelot and Falush 2007) as well as conventional maximum-likelihood (ML) methods.

## Materials and Methods

### Site description, soil sampling, and bacterial isolation

#### (a) Soybeans

Two field sites (A and B), about 280 km apart, were selected for soil sampling based on contrasting histories of soybean cultivation and inoculation. Site A consists of an experimental plot on the Central Experimental Farm (CEF), Ottawa, Ontario (latitude 45° 23′09.04″ N; longitude 75° 43′ 10.99″ W). Soybeans were cultivated at the CEF since 1897, but at site A, they were first grown and inoculated (either with soil from a soybean field or bacterial culture) in 1939; from about 1970, soybeans were grown without inoculation. The soil is fine sandy loam (Melanic Brunisol), pH 6.7 (water).

Site B is a farmer's field at St Hugues, Quebec (latitude 45° 50′ 25.84″ N; longitude 72° 52′ 03.63″ W). The soil is poorly drained clay loam (Orthic Humic Gleysol), pH 7.0 (water). This site had no history of soybean cultivation until 1992 when soybeans were introduced and inoculated (Nitragin Co., Milwaukee, WI); soybeans were subsequently grown and inoculated in each of 5 years with corn (*Zea mays*) cultivated in intervening years.

At the time of soil sampling (July 1999), soybean cultivar AC Maple Glen was grown at both sites; all plants examined were well nodulated. Thirty soil samples (15-cm depth) were collected with aseptic precautions from the vicinity of soybean roots, but otherwise at random, from a 25-m^2^ area at each site. Soil samples were pooled to form a composite for each site and maintained at 4°C before use.

Isolation of bradyrhizobia from soil samples A and B was done (within 7 days of soil sampling) using soybean cultivars AC Maple Glen and AC Orford (subsequently abbreviated M and O, respectively) as trap plants; both cultivars are short season soybeans used in eastern Canada. A soil suspension (fivefold dilution in water) of each soil sample was mixed for 20 min and 10-mL aliquots used to inoculate soybean seedlings from surface-sterilized seed planted in Leonard jars (Vincent 1970) containing vermiculite and supplied with nitrogen-free nutrient solution (Bromfield et al. 1995); controls consisted of uninoculated plants. Plants were maintained in a controlled environment chamber for 35 days at 25°C (16 h day) 17 °C (8 h night). Bradyrhizobia were isolated from surface-sterilized nodules (Bromfield et al. 1995) taken at random from roots of plants in 10 replicate jars (two plants per replicate) for each soil and cultivar combination; uninoculated plants were without nodules.

Bacteria were grown at 28°C using yeast-extract-mannitol (YEM) agar (Vincent 1970), modified to contain (gL^−1^): 1.5 yeast extract (Oxoid, Basingstoke, Hampshire, UK) and 1.0 mannitol; 0.15 cycloheximide (Sigma-Aldrich, Oakville, Ontario, Canada) was added to inhibit fungal growth for bacterial isolation. Bacteria were purified by streaking and single colony picking and maintained at −80°C in 20% (w/v) glycerol for subsequent analysis.

The number of viable bradyrhizobia in soil samples A and B was estimated by most probable number (MPN) method (Vincent 1970) using soybean cultivars M and O grown in pouches (Mega International, West St. Paul, Minnesota) and fivefold soil dilutions. The test was carried out in 1999 within 14 days of soil sampling.

#### (b) Native legumes

Thirty root-zone soil samples were collected with aseptic precautions (10-cm depth) from each of the native legumes, *D. canadense* (tribe *Desmodieae*) and *A. bracteata* (tribe *Phaseoleae*) growing in natural woodland habitats in Quebec: Aylmer (45°22′48.21″N 75°48′5.52″W) and Donnacona (46°41′04.83″N 71°44′28.05″W), respectively. These native legumes were chosen because they are fairly common and are distributed throughout the soybean growing regions of eastern Canada. Suspensions of composite soil samples representing each legume were prepared and aliquots inoculated onto seedlings of soybean cultivars O and AC Glengarry grown in Leonard jars. All procedures, including plant growth and bacterial isolation, were carried out as described in the preceding section. AC Glengarry replaced AC Maple Glen for use in east Canada in 2000 (Cober et al. 2007).

### Bradyrhizobia

Totals of 220 (from soybean field sites A and B) and 148 (from *D. canadense* and *A. bracteata*) bacterial isolates were analyzed. All bacterial isolates from soybeans (sites A and B) and native legumes were slow growing on YEM agar (colony diameter <1 mm after 7–21 days at 28°C).

Reference bradyrhizobia included eight strains known to have been used in Canadian commercial soybean inoculants, 10 named species (type strains) and the photosynthetic bacterium, *Bradyrhizobium* sp. BTAi1 (Table 1; Tables S2–S5).

**Table 1:** *Bradyrhizobium* strains used in soybean inoculants

Strain and alternative designation	Year introduced	Origin/Characteristics/Reference	ST	Core lineage/*nod*C group
61A101	1974, discontinued 1987*	Nitragin Co., Milwaukee, WI; isolated Illinois; serogroup C3	32	I/*nod* II
61A124	1974, discontinued 1986*	Nitragin Co., Milwaukee, WI; isolated New Zealand	NA	NA
532C (61A152, SEMIA 5039)	1990†‡	Isolated Brazil from soybeans inoculated with strains from the United States of America (Santos et al. 1999)	24	V/*nod* I
USDA138 (61A118, SEMIA 5028)	1974*	USDA/ARS, Beltsville, MD, isolated Mississippi, 1961; serogroup 6 (Keyser and Griffin 1987)	24	V/*nod* I
USDA 136 (CB1809, RCR3407, TAL379, 61A136, SEMIA 0586)	1980†‡	USDA/ARS, Beltsville, MD; reisolate of USDA122; serogroup 122 (Keyser and Griffin 1987)	4	IV/*nod* I
USDA122	See USDA136	USDA/ARS, Beltsville, MD; isolated Mississippi, 1960. Parent strain of USDA136; serogroup 122 (Keyser and Griffin 1987)	4	IV/*nod* I
USDA110 (3I1b110, TAL102)	1980†	USDA/ARS, Beltsville, MD; isolated Florida, 1959; serogroup 110 (Keyser and Griffin 1987)	9	IV/*nod* I
USDA142 (61A148, 3I1b142, SEMIA5058)	1974*	Isolated India, 1973; serogroup 122 (Keyser and Griffin 1987)	3	IV/*nod* I

Data are for inoculant strains used in Canada up to 1999 (the time of soil sampling and bacterial isolation); no information available for proprietary strains. NA, not recovered from field sites A or B. *Bradyrhizobium japonicum* USDA6^T^ has the same multilocus genotype as strain 532C. Strain 532C was supplied by T. Wacek, BeckerUnderwood/Urbana, strains 61A101 and 61A124 by the Nitragin Co, and the remaining strains by P. van Berkum, USDA/ARS.

Information source:

* S. Smith, Nitragin Co., Milwaukee, WI.

† T. Wacek, Becker Underwood/Urbana, St. Joseph, MO.

‡ D. Blair, Central Food Inspection Agency, Ottawa, Canada.

### Nucleotide sequencing

Partial sequences of six chromosomally encoded core genes (*atp*D, *gln*II, *rec*A, *gyr*B, *rpo*B, and *dna*K) were generated for the 220 bacterial isolates from sites A and B as well as for reference strains not available in Public databases. Partial *rec*A gene sequences were generated for all 148 bacterial isolates from native legumes together with partial *dna*K sequences for selected isolates. The six core genes used in this study were selected based on their relatively uniform distribution on the chromosomes of *B. japonicum* USDA6^T^ (Kaneko et al. 2011) and USDA110 (Kaneko et al. 2002) (Table S1) as well as their previous use in phylogenetic studies of *Bradyrhizobium* sp. (Vinuesa et al. 2008; Menna et al. 2009; Rivas et al. 2009).

To assess phylogenetic relationships based on genes located on the symbiosis island region of the bacterial chromosome, partial sequences of the *nod*C gene (encoding nodulation protein C involved in nodulation factor synthesis) were generated for all 220 isolates from field sites A and B as well as for selected isolates from native legumes; partial sequences of the *nif*H gene (encoding metalloprotein II of the nitrogenase enzyme responsible for biological nitrogen fixation) were generated for selected isolates.

Preparation of genomic DNA, amplification, nucleotide sequencing, and sequence editing was as described by Bromfield et al.(2010), except that amplifications were performed using a TProfessional thermocycler (Biometra, Goettingen, Germany) with 10-μL reaction mixtures containing 1–1.5 ng DNA, 0.1 mmol/L each dNTP, 0.08 μmol/L each primer, 0.5× Titanium *Taq* DNA polymerase (Clontech Laboratories Inc., Mountain View, California), and 1× buffer that was supplied with the enzyme.

Primers for amplification and sequencing were derived from the literature and, together with temperature and polymerase chain reaction (PCR) cycling conditions, are shown in Table S6. GenBank accession numbers of the 1779 nucleotide sequences generated in this study are listed in Tables S2–S5.

### Analysis of sequence data

Sequences were read in frame and aligned using ClustalW and RevTrans version1.4 (Wernersson and Pedersen 2003) taking into account corresponding amino acid alignments. Editing of alignments was done based on protein-encoding genes using Mega5 (Tamura et al. 2011); alignments were trimmed, so that sequences of each gene were the same length.

Core gene sequences that differed from each other by one or more polymorphisms were identified using the unique.seqs command implemented in Mothur 1.2 (Schloss et al. 2009). Sequences were concatenated using BioEdit 7 (http://www.mbio.ncsu.edu/bioedit/bioedit.html) and each unique allelic profile assigned a sequence type (ST) number.

### Population structure and ancestry

Population structure and ancestry of the 220 bacterial isolates from field sites A and B was inferred using the admixture model with independent allele frequencies (Pritchard et al. 2000) implemented in STRUCTURE version 2.3. Sequence data (six core genes) were formatted using xmfa2struct (http://www.xavierdidelot.xtreemhost.com/clonalframe.htm). STRUCTURE uses a Bayesian clustering framework and assumes that the observed data are derived from *K* ancestral populations (lineages). The admixture model allows for the possibility that individuals may have mixed ancestry in more than one of the *K* populations.

Five replicate Markov Chain Monte Carlo (MCMC) runs were performed for each value of *K* ranging from 2 to 7 using 100,000 burn-in and 200,000 sampling iterations. The ad hoc approach described in the software documentation was followed to select a value for *K* as well as the additional criterion that an ancestral population must contribute >50% genetic material to at least one individual to be recognized. Software CLUMPP (Jakobsson and Rosenberg 2010) was used to account for label switching in five replicate STRUCTURE runs of the selected *K*.

### Phylogenetic relationships between bradyrhizobia and reference strains

ML phylogenetic analyses were carried out using PhyML version 3.0 (Guindon et al. 2010) and the substitution model GTR+G+I, selected on the basis of the Akaike information criterion implemented in jMODELTEST version 0.1 (Posada 2008). Settings used in PhyML were as follows: five random starting trees, estimated gamma shape parameter (four substitution rate categories), estimated proportion of invariable sites, and SPR and NNI tree improvement algorithms. ML trees were reconstructed using unique STs for concatenated core gene sequences and unique sequences for symbiotic genes; 1000 nonparametric bootstrap replications were used to assess support. Trees were drawn using Mega5 software.

Data for 1000 replicate bootstrap trees from the ML analysis of six concatenated core gene sequences were imported into SplitsTree version 4.1 (Huson and Bryant 2006) and used to construct a consensus network graph.

A randomization test was carried out to assess the extent of congruence between ML trees reconstructed for each of the six core genes employing custom R scripts (R Development Core Team 2009) based on the phangorn phylogenetic package (Schliep 2011). This test assesses whether ML trees for different genes are more similar to each other than to trees of random topology (Feil and Spratt 2001). One hundred random trees were generated, and, following the method of Feil and Spratt (2001), 11 unique STs were selected to represent the different STRUCTURE lineages from a tree inferred by ClonalFrame (see below). An example of R code used in these calculations is given in Table S7.

### ClonalFrame analysis of recombination

To further investigate microevolutionary relationships and to infer recombination events, 50% majority rule consensus trees were computed for sequence data of the six core genes using ClonalFrame version 1.2 (Didelot and Falush 2007).

ClonalFrame employs a Bayesian framework to infer clonal relationships while taking into account recombination.

Five independent MCMC runs, with and without correction for recombination, were performed, using sequence data for 220 bacterial isolates from field sites A and B. Each run consisted of one million iterations. The first 500,000 iterations of each run were discarded and model parameters sampled every 100 generations, thereafter producing a sample size of 5000 from the posterior. Convergence of the MCMC was judged satisfactory based on the Gelmin–Rubin test and genealogy comparison tool implemented in the Graphical User Interface.

Recombination events were recognized when the posterior probability of an import was above 95%. Measures of recombination rate computed were as follows: ρ/θ, the ratio of recombination and mutation rates; and r/m, the ratio of probabilities that a given nucleotide will be altered through recombination and mutation.

### Population genetics analysis

DnaSP version 5.1 (Librado and Rozas 2009) was used to calculate summary statistics for sequences representing the 220 bacterial isolates from field sites A and B. Statistics calculated included G+C content, the number of polymorphic (segregating) sites (*S*), and the haplotype (gene) diversity (Hd). The average number of pairwise nucleotide differences per site (π), number of synonymous substitutions per synonymous site (π_S_), number of nonsynonymous substitutions per nonsynonymous site (π_N_), and the ratio of nonsynonymous to synonymous substitutions (d*N*/d*S*) were calculated with Jukes–Cantor correction. Tajima's *D* test of neutrality was calculated based on segregating sites. The following statistics of genetic differentiation and gene flow were calculated: average number of nucleotide substitutions per site between populations (*Dxy*), the sequence-based statistic of genetic differentiation (*K*_ST_*) (Hudson et al. 1992a) with permutation tests (10,000 replications) to assess statistical significance, the effective number of migrants (*Nm*) and the fixation index (*F*_*ST*_) described by Hudson et al. (1992b). The codon-based *Z*-test of purifying selection implemented in Mega5 was carried out using the Pamilo–Bianchi–Li method, pairwise deletion, and 500 bootstrap replications.

### Relative effectiveness

Relative nitrogen-fixing effectiveness (RE) of bacterial isolates was assessed using soybeans grown in Leonard jars (Vincent 1970) according to methods and conditions described by Bromfield et al. (2010). After 35 days, shoots were removed and dried to constant weight at 80°C.

RE of bacterial isolates was calculated as ([*x* − *x*^o^]/[*x*^e^ − *x*^o^]) × 100, where *x*, *x*^o^, and *x*^e^ are the mean shoot dry weights of, respectively, plants inoculated with a given bacterial isolate, uninoculated plants, and plants inoculated with an effective reference strain. RE values were derived from means of five replicates (two plants/replicate) for each bacterial isolate.

## Results

### Description of *Bradyrhizobium* populations at field sites A and B

Details of the origin (field site, soybean cultivar) of the 220 bacterial isolates are listed in Table S2. The MPNs of viable symbiotic bacteria were averaged over two soybean cultivars used as trap hosts. MPN values (×10^5^ g^−1^ soil) were 8.5 (site A) and 30 (site B) indicating large census population sizes of soybean-nodulating bacteria at both field sites at the time of soil sampling.

The six concatenated core gene sequences (220 isolates) were classified into 35 unique STs. Isolates representing totals of 15 and 27 STs were recovered from sites A and B with seven STs common to both field sites (Table 2). At site A, isolates of ST15 and ST21 were predominant and accounted for ∼62% isolates at this site. At site B, isolates of six STs accounted for ∼61% of the isolates.

**Table 2:** Frequency and relative effectiveness (RE) of isolates representing 35 STs from field sites A and B

			No. isolates from					
					
			Site A		Site B			
						
			Soybean cultivar					
Lineage	ST	*nodC* group	M	O	M	O	Frequency	RE (Isolate No.)
I	31	*nod* II				2	0.009	60 (HO186)
I	**32**	*nod* II			3	7	0.046	52 (HO199)
II	14	*nod* I			16		0.073	–
II	15	*nod* I	13	15	5	3	0.164	52 (OO107)
II	16	*nod* I				1	0.005	–
II	17	*nod* I				1	0.005	–
II	18	*nod* I	1				0.005	–
II	19	*nod* I				1	0.005	–
II	20	*nod* I	3				0.014	–
II	21	*nod* I	24	17	11	1	0.241	126 (OM55)
II	22	*nod* I				7	0.032	–
II	23	*nod* I			1		0.005	–
III	33	*nod* I		7			0.032	0 (OO85)
III	34	*nod* I	3	5			0.036	20 (OM9)
III	35	*nod* I		1			0.005	136 (OO99)
IV	1	*nod* I				2	0.009	–
IV	2	*nod* I				1	0.005	97 (HO190)
IV	**3**	*nod* I			10	11	0.096	152 (HO185)
IV	**4**	*nod* I	1				0.005	123 (OM50)
IV	5	*nod* I				1	0.005	–
IV	6	*nod* I				2	0.009	–
IV	7	*nod* I	2		2	2	0.027	60 (OM17)
IV	8	*nod* I			4		0.018	–
IV	**9**	*nod* I			1	1	0.009	76 (HM155)
IV	10	*nod* I				1	0.005	–
IV	11	*nod* I	5			4	0.041	22 (OM15)
IV	13	*nod* I	1		2		0.014	–
V	12	*nod* I				1	0.005	–
V	**24**	*nod* I				1	0.005	7 (HO196)
V	25	*nod* I				1	0.005	124 (HO172)
V	26	*nod* I				1	0.005	–
V	27	*nod* I	1	7		1	0.041	127 (OO61)
V	28	*nod* I	1			2	0.014	63 (OM28)
V	29	*nod* I		1			0.005	–
V	30	*nod* I		2			0.009	–

Bacterial isolates were from soybean cultivars Maple Glen (M) and AC Orford (O). *nod*C groups were inferred by ML analysis. STs shown in bold share the same multilocus genotype as inoculant strains.

*Bradyrhizobium* strains known to have been used in soybean inoculants up to 1999 (the time of soil sampling/bacterial isolation) are shown in Table 1. Strain USDA136 had the same ST as strain USDA122 (ST4); 532C and USDA138 also had an identical ST to *B. japonicum* USDA6^T^ (ST24). These inoculant strains (classified as ST4 and ST24) are subsequently referred to as USDA136 and 532C, respectively.

A minority of isolates recovered from both field sites exhibited the same STs as inoculant strains. At site A, only one isolate (ST4) had the same genotype as an inoculant strain (USDA136), but it is unlikely that this isolate originated from inoculation because soybeans were grown without inoculation at site A when USDA136 was brought into service in 1980 (Table 1). At site B, ∼31% isolates had identical STs to inoculant strains. These were one isolate of ST24 (532C), two isolates of ST9 (USDA110), 21 isolates of ST3 (USDA142), and 10 isolates of ST32 (61A101). As inoculant strain 61A101 (ST32) was discontinued in 1987, several years before soybeans were cultivated at site B, it is highly unlikely that isolates of ST32 originated from inoculation. For similar reasons, it was not surprising that isolates with the same ST as inoculant strain 61A124 (years of service, 1974–1986) were not encountered at either field site (Table 1). Adjusting for years of service of inoculant strains and inoculation history, isolates of putative inoculant strains were not recovered from site A, whereas about 20% were recovered from site B.

### Summary statistics

Summary statistics for nucleotide sequence data (220 isolates, field sites A and B) are shown in Table 3. There were seven allelic types (*h*) for the symbiotic *nod*C gene and between 9 (*dna*K) and 17 (*rpo*B) for core genes. The lower G–C content of the *nod*C gene (57%) relative to the average of the six core genes (65%) is consistent with the hypothesis of ancient symbiosis island integration into the *Bradyrhizobium* chromosome following acquisition from an external source (Kaneko et al. 2011).

**Table 3:** Summary statistics for core and symbiotic (*nod*C) partial gene sequences of 220 bacterial isolates from field sites A and B

Locus	Sequence length (bp)	GC content (%)	*h*	*S*	π	π_S_	π_N_	d*N*/d*S*	*Z*	*D*
Core gene										
*atpD*	435	65.3	10	43	0.0258	0.0778	0.0095	0.122	4.18	1.502
*glnII*	555	63.5	16	91	0.0288	0.0997	0.0097	0.097	5.27	-0.008
*recA*	462	67.3	11	73	0.0353	0.1404	0.0025	0.018	6.60	0.836
*gyrB*	618	64.0	10	88	0.0343	0.1199	0.0097	0.081	6.19	1.182
*rpoB*	771	63.9	17	111	0.0300	0.1033	0.0084	0.082	7.11	0.609
*dnaK*	369	66.0	9	50	0.0309	0.1127	0.0092	0.082	4.03	0.898
Concatenated	3210	64.8	35	456	0.0308	0.1077	0.0083	0.077	12.39	0.791
Symbiotic gene										
*nod*C	726	57.3	7	118	0.0188	0.0763	0.0058	0.076	7.32	-1.191

*h*, number of haplotypes (alleles); *S*, number of polymorphic (segregating) sites; nucleotide diversity estimated for all sites (π), synonymous sites (π_S_), and nonsynonymous sites (π_N_) with Jukes–Cantor correction; d*N*/d*S*, ratio of nonsynonymous to synonymous substitutions; *Z*, codon-based test of purifying selection (500 bootstrap replications); all values are significant (*P* = 0.000); *D*, Tajima's *D* based on segregating sites; all values are not significantly different from 0 (*P* > 0.1).

Values of *dN*/*dS* were <1 indicating that all loci were subjected to purifying selection. This conclusion is supported by significant (*P* = 0.000) values of the *Z*-test statistic rejecting the null hypothesis of *d*_*N*_ = *d*_*S*_. Strong purifying selection is consistent with essential functions of core genes and with the functions of the *nod*C gene generating signal molecules essential for initiation of plant–bacterial symbiosis. Tajima's *D* values were not significantly different from 0 for all loci suggesting no significant departure from a standard neutral model with purifying selection (Feil 2010).

### Population structure and ancestry

Different STRUCTURE models were explored with *K* (number of ancestral lineages) ranging from 2 to 7. Data for multiple STRUCTURE runs using the admixture model with independent allele frequencies (220 bacterial isolates, sites A and B) indicated that *K* = 5 was optimal based on criteria described in Methods; at values of *K* between 2 and 5, each additional *K* contributed up to 100% genetic material to multiple isolates, whereas at values of *K* between 6 and 7, each new *K* contributed <10% genetic material only to a single isolate of ST24 (532C). Although the marginal likelihood plateaued at *K* = 5 (Table S8), this may be coincidental given known problems with the harmonic mean estimator (Lartillot and Philippe 2006).

**Figure 1:**
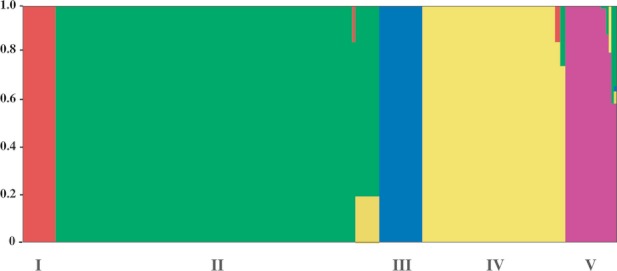
Core gene ancestries of 220 *Bradyrhizobium* isolates from field sites A and B inferred by STRUCTURE. Proportions of ancestry from lineage I (red), II (green), III (blue), IV (yellow), and V (magenta) was inferred assuming *K* = 5 ancestral populations. Each isolate is represented by one vertical line color coded according to the proportion of single nucleotide alleles that each isolate derived from one of the ancestries.

Data for ancestry and admixture levels of the 220 isolates are shown in Figure 1. Isolates with >50% genetic material from one of the five ancestral lineages are considered to be representative of that lineage. Among the sample of isolates, those assigned to lineage II were most abundant (120 isolates), whereas those in lineage I (12 isolates) were the least. Isolates of lineage I and III were each encountered at one of two sites and had 100% of their genetic material derived from the respective ancestral lineage. The majority of isolates in lineages II, IV, and V (encountered at both field sites) exhibited ancestries that were homogeneous. Of 172 isolates representing these three lineages, only 20 isolates (12 STs) exhibited mixed ancestries suggesting that limited interlineage flow of core genes had taken place. The isolate representing ST24 (532C) in lineage V was unusual in that it was highly admixed, possessing genetic material inherited from four ancestral lineages. All 20 isolates with mixed core gene ancestries were from soybean cultivar O. This appears consistent with data indicating higher levels of haplotype, nucleotide, and gene diversity (core and *nod*C gene sequences) for isolates from soybean cultivar O relative to cultivar M (Table S9).

### Phylogenetic relationships between bradyrhizobia from field sites A and B and reference strains

**Figure 2:**
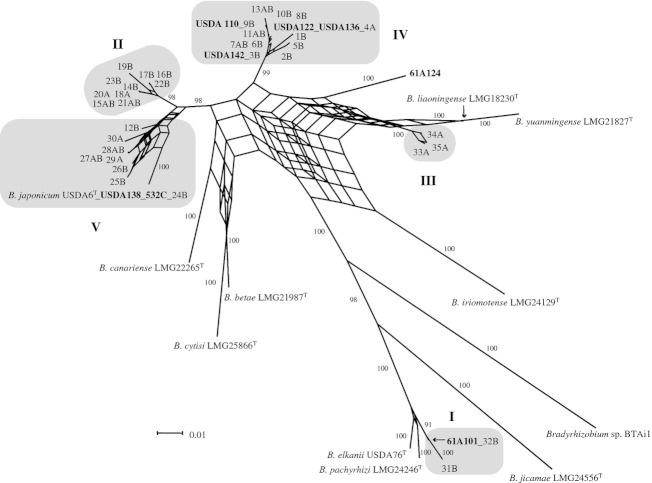
Consensus network graph of *atp*D-*gln*II-*rec*A-*gy*rB-*rpo*B-*dna*K concatenated gene sequences (3210 bp) for *Bradyrhizobium* reference strains and 35 unique STs representing 220 bacterial isolates from field sites A and B. Consensus network based on 1000 replicate bootstrap trees from ML analysis. Values on graph edges indicate confidence. Letters A, B, and AB following ST numbers designate site of origin. STs connected by underscores have the same ST as indicated reference strains; inoculant strains are shown in bold. Gray shaded clusters and roman numbers designate isolates in lineages inferred by STRUCTURE. Lineages V and IV correspond to *B. japonicum* groups 1 and 1a, respectively; lineages I, II, and III are novel. Superscript T designates type strains; scale bar = 1% substitutions per site.

The consensus network graph based on 1000 replicate bootstrap trees from ML analysis of concatenated core gene sequences representing isolates from sites A and B and reference strains is shown in Figure 2; the corresponding ML tree is shown in Figure S1.

Using a relatively low value of 0.1 for the consensus network threshold, moderate reticulation was evident, particularly on inner edges. Reticulation reflects phylogenetic uncertainty and may indicate recombination.

The network graph and corresponding ML tree show well-defined clusters of isolates corresponding to the five lineages inferred by STRUCTURE.

Graph edges within clusters corresponding to lineages I, II, and III show little reticulation. These clusters have no type strain representatives indicating that they are novel evolutionary lineages within the genus *Bradyrhizobium*.

Lineage I was part of a clade with *B. elkani* and *B. pachyrhizi*. The closest relative of lineage II isolates was lineage V, whereas the nearest neighbors of lineage III isolates were *B. liaoningense* and *B. yuanmingense*.

Little reticulation was also evident in the cluster of isolates representing lineage IV that included inoculant strains USDA110, USDA136, and USDA142. In contrast, the lineage V cluster containing *B. japonicum* USDA6^T^ showed moderate reticulation. Lineages V and IV correspond to “*B. japonicum*” groups 1 (USDA6^T^) and 1a (USDA110) defined on the basis of DNA homology (Hollis et al. 1981). Inoculant strain 61A124 was not recovered from field sites A or B, but was placed in a distinct lineage.

**Figure 3:**
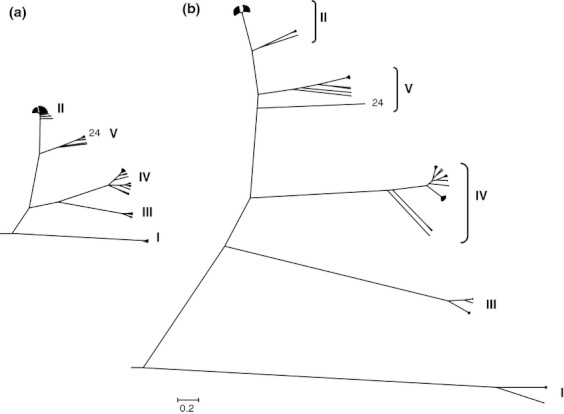
Majority rule consensus trees inferred by ClonalFrame showing the effect of recombination on branch length and branching order. Trees are with (a) and without (b) correction for recombination and share the same scale in coalescent units. Data are for six core genes representing 220 bacterial isolates from sites A and B. Roman numbers designate STRUCTURE lineages I to V. The placement of ST24 is indicated.

Randomization tests were used to assess the extent of congruence between ML trees reconstructed for each of the six core genes. The 11 STs (2, 4, 9, 11, 12, 15, 16, 25, 30, 31, and 35) used in the analysis were selected to represent the different lineages in a ClonalFrame tree (Fig. 3a). Relative to 100 randomly generated trees, only the *gyr*B gene tree was significantly incongruent with trees of the other five genes irrespective of whether reference strains were included in the analysis (Fig. S2). Moreover, a ML tree (reconstructed for all unique *gyr*B sequences; Fig. S4) was markedly incongruent with the ML “species” tree of concatenated gene sequences (Fig. S1), suggesting that extensive intragenic recombination had occurred at the *gyr*B locus: a minimum of 10 lateral transfer events were inferred for STs in lineages I, II, IV, and V.

### ClonalFrame analysis of microevolution and recombination

Microevolution and the role of recombination in genetic diversification were further investigated by ClonalFrame analysis of all 220 isolates from sites A and B.

To investigate the effect of recombination on branch length and branching order, trees were compared with and without correction for recombination (Fig. 3). Time (path length in coalescent units) to the most recent common ancestor (TMRCA) of all isolates was calculated for each tree. Based on five independent runs, TMRCA values were 1.33 with 95% credibility interval (CI) (1.00–1.70) for the recombination corrected tree (Fig. 3a) and 3.84 with CI (3.47–4.23) for the tree without correction for recombination (Fig. 3b) indicating that the TMRCA of all lineages was almost three times shorter when allowance was made for recombination. Moreover, the branching order of the two trees differed markedly. For example, Figure 3a shows that the isolate of ST24 is placed within lineage V, but in Figure 3b, it is not. Similarly, lineages III and IV are monophyletic in Figure 3a, whereas in Figure 3b, they are not.

**Figure 4:**
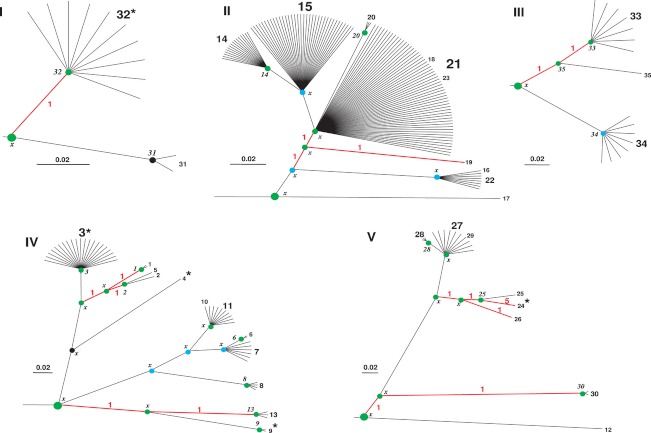
Distribution of recombination events on branches of sub-trees representing the five STRUCTURE lineages in Figure 3a. Lineages are labelled I–V. Font sizes of ST numbers on leaves are approximately proportional to the number of isolates represented. Asterisks denote STs with the same multilocus genotype as reference inoculant strains (see Table 1). STs on internal nodes are inferred ancestors and are italicized; *x* indicates that the ST was not present in the sample. Red numbers indicate the number of recombination events with posterior probability above 95% on branches highlighted in red. Confidence values (%) for internal nodes are: green (90–100), blue (70–90), and black (less than 70). Scale bars represent coalescent units.

The ratio of recombination events relative to mutation (ρ/θ) estimated for the 220 isolates (five replicate runs) was 0.05 with CI (0.02–0.08) indicating that recombination was considerably less frequent than mutation. The effect of recombination relative to mutation (r/m) was 0.59 with CI (0.34–0.88) indicating that recombination introduced almost two times fewer substitutions than mutation. Because clonal expansion of specific bacterial genotypes, as observed for lineage II in this study (Fig. 4), may result in underestimation of recombination rates (Smith et al. 2000), the ρ/θ and r/m statistics were inferred in a separate analysis using unique STs only. Consistent with expectations, recombination rates based on STs only (ρ/θ = 0.09 with CI [0.04–0.14] and r/m = 1.00 with CI [0.59–1.53]) were higher than those inferred for all isolates.

The tree in Figure 3a was analyzed to assess the distribution of recombination events (probability above 95%) on branches of sub-trees each representing one of the five lineages. Results are shown in Figure 4 and events found at each locus are listed in Table 4. A total of 21 events were found in the five sub-trees (Fig. 4). One recombination event was found in lineage I, three in lineage II, two in lineage III, five in lineage IV, and 10 in lineage V. These levels of recombination are consistent with the extent of intralineage reticulation in the network graph (Fig. 2) and with the average levels of nucleotide diversity (concatenated core genes) of lineages II (0.0018), IV (0.0043), and V (0.0074) (Table 5). Twelve recombination events were found at the *gyr*B locus, whereas only between one and three were found at each of the remaining loci. These data are in agreement with the phi test (Huson and Bryant 2006) that detected significant recombination (*P* = 0.002) at the *gyr*B locus, with results of the congruence test (Fig. S2), and with higher levels of nucleotide diversity (lineages II, IV, and V) at the *gyr*B locus than at other loci (Table 5). The isolate of ST24 (lineage V), that was highly admixed, exhibited five events at *atp*D, *gln*II, *rec*A, *rpo*B, and *dna*K loci on the external branch from ST24 to the MRCA providing further evidence of recombination in the history of ST24 (Fig. 4).

**Table 4:** Number of recombination events inferred by ClonalFrame analysis of Figure 4

	Core gene						
		
Lineage	*atp*D	*gln*II	*rec*A	*gyr*B	*rpo*B	*dna*K	Total
I	0	0	0	1	0	0	1
II	0	0	0	3	0	0	3
III	0	1	0	0	1	0	2
IV	0	0	2	3	0	0	5
V	1	1	1	5	1	1	10
Total	1	2	3	12	2	1	21

Lineages I–V inferred by STRUCTURE. Only events with a posterior probability of 95% or greater are recognized.

**Table 5:** Nucleotide diversity of selected *Bradyrhizobium* lineages from field sites A and B

	Lineage II (*n* = 120)			Lineage IV (*n* = 53)			Lineage V (*n* = 19)		
			
Gene	*h*	*S*	π	*h*	*S*	π	*h*	*S*	π
Core genes									
*atp*D	1	0	0	2	1	0.00033	4	16	0.00588
*gln*II	3	2	0.00093	5	5	0.00209	5	15	0.00431
*rec*A	1	0	0	6	18	0.00832	2	9	0.00208
*gyr*B	5	57	0.00814	5	56	0.00934	5	58	0.02202
*rpo*B	3	2	0.00037	6	13	0.00319	5	14	0.00393
*dna*K	1	0	0	2	1	0.00138	4	9	0.00383
Concatenated	10	61	0.00177	12	94	0.00426	8	121	0.00739
Symbiotic gene									
*nod*C	2	1	0.00002	2	1	0.00070	3	2	0.00066

*n*, number of sequences. For explanation of other symbols see Table 3.

The topology of the sub-tree representing lineage II (Fig. 4) suggests that a recent clonal expansion of STs 15 and 21 had taken place. To further investigate this issue, an external/internal branch length ratio test was performed on the recombination corrected tree generated from a ClonalFrame analysis (5 replicate runs) of the lineage II data (*n* = 120). As the lineage II dataset was smaller than the combined dataset, the mean length of an import was fixed at the value computed for the whole dataset. The results (Fig. S3) indicate that the tree of lineage II was unexpectedly star-shaped with a ratio that was significantly higher than expected based on coalescent simulations (0.66, *P* = 0.007), consistent with a contemporary clonal expansion (partial selective sweep).

Further analysis was done to estimate the TMRCA of lineage II relative to lineages IV and V from the recombination corrected tree computed for all isolates (Fig. 3a). The TMRCAs (based on branch lengths) of lineages IV and V were similar, but about twice that of lineage II. This suggests that lineages IV and V may have appeared at about the same time, whereas lineage II may have emerged more recently. The exceptionally low nucleotide diversity at most loci of lineage II (Table 5) relative to lineages IV and V appears consistent with an inferred recent origin of this lineage, with a relatively low level of recombination and with the diversity purging effect of a partial selective sweep.

The extent of genetic differentiation and divergence between lineages II, IV, and V was further analyzed using population subdivision and gene flow statistics (Table S10). Based on concatenated core gene sequences, the high values of population subdivision statistics (*Dxy*, *K*_ST_*, and *F*_*ST*_), the high number of fixed differences, and the low effective number of migrants (*Nm*) all indicate substantial differentiation and genetic isolation of lineages II, IV, and V. These statistics suggest that the extent of genetic divergence and isolation was greatest for lineages II versus IV, IV versus V, and least for sister lineages, II versus V, consistent with the relative placement (branch lengths) of these lineages in the recombination corrected genealogy (Fig. 3a).

### Relationships between bradyrhizobia associated with soybeans and native legumes

Using two soybean cultivars as trap plants, 148 isolates of soybean-nodulating bacteria were obtained from root-zone soils of native legumes, *A. bracteata* (29 isolates) and *D. canadense* (119 isolates). Relatively few nodules (between 7 and 25 per plant) were elicited on roots of trap plants. Symbiotic bacteria associated with these native legumes varied with regard to nitrogen-fixing effectiveness on soybeans, as judged by variation in nodule size (2–10 mm), interior nodule color (white/green to pink), and low to intermediate overall relative effectiveness (RE) values (Table 6).

**Table 6:** Characteristics of symbiotic bradyrhizobia from root-zone soils of *Amphicarpaea bracteata* and *Desmodium canadense* using soybeans as trap plants

	Trap host (soybean cultivar)				
		
	AC Glengarry		AC Orford		
			
Root-zone soil from native legume	Ave. nodules per plant	RE	Ave. nodules per plant	RE	Core lineage/*nod*C-*nif* H group (no. of isolates)
*A. bracteata*	16.7	5.2	6.8	16.3	Lineage I/*nod*-*nif* II (10); Lineage II/*nod*-*nif* I (19)
*D. canadense*	24.7	32.9	24.0	51.4	Lineage V/*nod*-*nif* I (119)

Relative effectiveness (RE) values are based on uninoculated plants supplied with 1% (w/v) KNO3 instead of an effective reference strain. Lineage assignment based on analysis of the *rec*A (148 isolates), *dna*K, *nod*C, and *nif* H (selected isolates) partial gene sequences shown in Tables S4 and S5.

Phylogenetic trees of *rec*A (all isolates) and concatenated recA-*dna*K (selected isolates) partial gene sequences representing bradyrhizobia from native legumes and soybean sites A and B are shown in Figure S5. Soybean-nodulating bacteria from *D. canadense* exhibited the same *rec*A-*dna*K genotype as lineage V isolates (*B. japonicum* group I); two of these isolates had the same genotype as inoculant strain 532C and type strain, USDA6^T^ (ST24). In contrast, all sequences of soybean-nodulating bacteria from *A. bracteata* clustered with bacteria in lineages I and II.

Plant tests with *A. bracteata* showed that 8 of 10 and 9 of 10 plants were nodulated following inoculation with suspensions of soil from field sites A and B, respectively (data not shown). Although soybean-nodulating bacteria in lineages III and IV were not isolated from the two native legumes used in this study, results of plant tests showed that isolates representing lineage III (OO99) and lineage IV (HM155 and USDA110) from soybean field sites were capable of eliciting nodules on both *D. canadense* and *A. bracteata* (data not shown). Moreover, database searches showed that several *rpo*B (partially overlapping) and *dna*K sequences of bradyrhizobia from diverse legumes native to North America had 98–99% identity with sequences of isolates representing each of the five lineages defined in this study.

**Figure 5:**
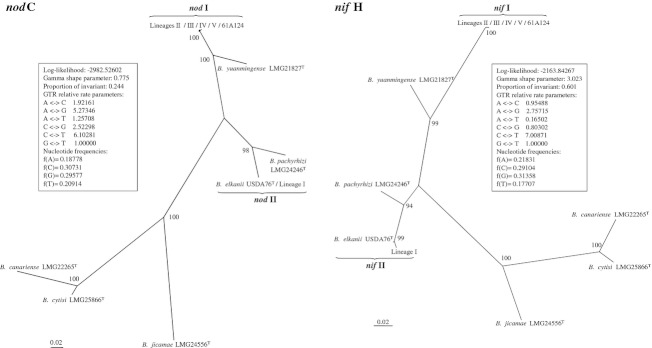
ML phylogenetic trees of *nod*C (726 bp) and *nif*H (669 bp) partial gene sequences representing reference taxa, soybean-nodulating bacteria from field sites A and B and from native legumes (*Amphicarpaea bracteata* and *Desmodium canadense*). The *nod*C phylogeny is based on unique sequences representing 220 bacterial isolates (sites A and B) and reference strains shown in Figure 2. The *nif*H phylogeny is based on reference strains and selected isolates. Both *nif*H and *nod*C trees include representative isolates from *A. bracteata* in lineages I and II and from *D. canadense* in lineage V (see Table S5 for isolate/sequence accession numbers). Bootstrap values >50% (1000 nonparametric replications) are indicated at nodes. Scale bar represents estimated substitutions per site.

ML gene trees of unique *nod*C and *nif*H partial gene sequences representing bacterial isolates from sites A and B and isolates from *A. bracteata* and *D. canadense* are shown in Figure 5. The topologies of these symbiotic gene trees are highly congruent consistent with ‘hitchhiking’ of *nod*C and *nif*H genes that are located in close proximity on the chromosomal symbiosis island region (Table S1) triggered by periodic selection events favoring particular adaptive symbiotic gene variants. While we were unable to generate a *nod*C sequence fragment (726 bp) of the soybean-nodulating bacterium, *B. liaoningense* LMG18230^T^, a partially overlapping sequence, available in Public databases (JN993965), showed 99% identity with *nod*C sequences of *B. japonicum* USDA6^T^ (AP012206) and USDA110 (BA000040) in the *nod* I group; a *nif*H sequence of LMG18230^T^ (EU818925) was placed in the *nif* I group (not shown).

Comparison of the topologies of the ML symbiotic gene trees with the network graph and ML tree of core genes (Fig. 2; Fig. S1) indicated that they were not in complete agreement, although it should be noted that the lack of resolution of inner edges in the network graph was a confounding factor.

In both symbiotic gene trees, sequences of soybean-nodulating bacteria from sites A and B and from native legumes were placed in two highly supported groups (*nod*-*nif* I and *nod*-*nif* II). The *nod*-*nif* I group included all soybean-nodulating bacteria in lineages II, III, IV (*B. japonicum 1a*), and V (*B. japonicum 1*) together with the lineage defined by inoculant strain 61A124, whereas the *nod-nif* II group was defined by *B. elkani* and lineage I. This nonrandom clustering of core gene lineages in symbiotic gene groups may indicate that the inheritance of symbiotic gene variants is predominantly by vertical transmission. Indeed, the phi test and RDP version 4 (Martin et al. 2010) suite of recombination detection methods (RDP, GENECONV, BootScan, Chimaera, MaxChi, SiScan) did not detect significant intragenic recombination at either *nif*H or *nod*C loci of isolates from sites A and B, native legumes, and reference taxa of soybean-nodulating bacteria. The substantially lower levels of nucleotide diversity at *nod*C relative to core gene loci of lineages II, IV, and V (Table 5) might also be a further indication of low levels of recombination at the *nod*C locus and/or a recent origin of this gene. Moreover, the recombination-corrected genealogy of core loci (Fig. 3a) suggests that lineages II, III, IV, and V (in *nod*-*nif* group I) are monophyletic consistent with a hypothesis of predominantly vertical transmission of symbiotic genes.

Tests were done to assess the relative nitrogen-fixing effectiveness (RE) of 17 isolates representing lineages I to V from field sites A and B using soybean cultivar O as test plant (Table 2). These isolates exhibited considerable variation in effectiveness relative to reference strain USDA110 and uninoculated plants. For example, RE values of isolates in lineage III varied between 0% (isolate OO85) and 136% (isolate OO99). Similarly, isolates in lineage II that were found to be associated with *A. bracteata* and predominated at both soybean sites varied between poorly effective (RE, 52%) for isolate OO107 and highly effective (RE, 126%) for isolate OM55. Isolate HO185 (lineage IV) that has a multilocus genotype corresponding to inoculant strain USDA142 was the most effective isolate tested (RE, 152%). Several isolates that had the same genotype as an inoculant strain were less effective than reference strain USDA110. In particular, isolate HO196 (ST24, lineage V) was ineffective (RE, 7%) and possessed the same core and symbiotic genotype as the effective inoculant strain 532C and two isolates of soybean-nodulating bacteria from *D. canadense*. Similarly, isolate HO186 (ST32, lineage I) from site B that was poorly effective (RE, 52%) had the same genotype as 10 isolates from *A. bracteata* as well as effective commercial strain 61A101 that was taken out of service before soybeans were grown at site B (Table 1). Isolate HM155 (lineage IV) with the USDA110 genotype was also less effective than the culture of USDA110 used as reference in these tests.

## Discussion

### Soybeans are colonized by bradyrhizobia originating from native legumes

In this study, the use of MLST of six core gene sequences as well as the combination of STRUCTURE, ClonalFrame, and conventional ML phylogenetic analyses indicated that the *Bradyrhizobium* populations associated with soybeans at two field sites (A and B) with contrasting histories of cultivation and inoculation were highly structured: all analyses supported division of 220 bacterial isolates in five lineages corresponding either to *B. japonicum* groups 1 (lineage V) and 1a (lineage IV) or to one of three novel lineages (I, II, and III) within the genus *Bradyrhizobium*.

Consistent with expectations based on early reports of variable, but often poor persistence of introduced strains in the years succeeding soybean inoculation (reviewed by Keyser and Li 1992 and Streeter 1994), none of the isolates from site A and about 20% from site B (the only site where soybeans had been inoculated recently) were found to have originated from inoculation sources. Despite a history of recurrent *Bradyrhizobium* inoculation at site B, only isolates with multilocus genotypes (ST) corresponding to USDA110, USDA136, and USDA142 were attributed to inoculation sources, and of these, only USDA142 showed evidence of clonal expansion further emphasizing that most introduced strains lacked traits for successful establishment and were rapidly purged from the legume crop ecosystem.

A study in the genus *Mesorhizobium* indicated that the appearance of competitive symbiotic bacteria following the introduction of an exotic crop legume and inoculant strain into a new environment was due to resident soil bacteria acquiring a symbiosis island from the introduced strain by lateral transfer (Nandasena et al. 2007). Our data for the genus *Bradyrhizobium* provide an alternative explanation for the rapid displacement of inoculant strains by resident soil bacteria and strongly suggest that soybeans were preferentially using bacterial symbionts that possessed the pre-existing capacity to nodulate the exotic host and originated from legumes native to eastern Canada. This was based on phylogenetic analyses of two core (*rec*A and *dna*K) and two symbiotic (*nif*H and *nod*C) gene sequences that placed soybean-nodulating bacteria from *A. bracteata* and *D. canadense* in the same core and symbiotic gene lineages as isolates encountered at soybean field sites. In particular, isolates in core lineage II were distinct from named species and inoculant strains, predominated at soybean sites and were associated with *A. bracteata*. The contemporary clonal expansion of this lineage, as evidenced by the topology of the recombination-corrected genealogy and significant external to internal branch length ratio test (Fig. 4 and Fig. S3), was dominated by a few closely related genotypes suggesting that these bacteria, in contrast to most inoculant strains, possessed symbiotic fitness or adaptive characteristics and were selected by the host plant (i.e., a partial selective sweep).

Although we did not recover soybean-nodulating bacteria in core lineages III and IV (*B. japonicum* 1a) from native legumes, plant infection tests indicated that isolates representing these lineages from soybean field sites were capable of nodulating both *A. bracteata* and *D. canadense*. Moreover, legumes native to east North America (*Apios americana* and *Desmodium glutinosum*) were reported to harbor rare genotypes similar to USDA110 (lineage IV) based on 16S and 23S rRNA sequence analysis (Parker 1999).

Interestingly, two isolates from *D. canadense* and 10 isolates from *A. bracteata* had the same core and symbiotic genotypes as, respectively, inoculant strains 532C (*B. japonicum* 1; lineage V) and 61A101 (lineage I; in a clade with *B. elkani*). Strain 61A101 was originally isolated from soybeans grown in the United States (Table 1), whereas 532C was isolated from soybeans grown in Brazil that were inoculated with strains from the United States (Santos et al. 1999), suggesting that both of these strains might have originated from legumes native to North America. Further support for this proposition was provided by our finding that a minority of isolates from field site B had the same ST and symbiotic genotype as, respectively, 532C and 61A101, but exhibited poorly effective nitrogen-fixing phenotypes atypical of inoculant strains that are invariably selected for high symbiotic effectiveness with soybeans. Moreover, inoculant strain 61A101 was taken out of service several years before soybeans were cultivated at site B (the only site where isolates with the 61A101 genotype were encountered), further indicating that these isolates originated from bacterial symbionts that were associated with native legumes.

Soybeans grown in the United States are often nodulated by resident soil bacteria exhibiting variable and sometimes sub-optimal nitrogen-fixing capabilities relative to inoculant strains (Kvien et al. 1981; Keyser and Li 1992). Our data are consistent with these reports in that they provide an indication of considerable variation in the nitrogen-fixing capabilities of soybean-nodulating bacteria associated with native legumes as well as among 17 isolates representing different core and symbiotic gene lineages from soybean sites. In particular, our finding that some isolates representing lineages of indigenous origin (e.g., lineage II) were more effective than a reference inoculant strain has important practical implications and suggests that bradyrhizobia associated with native legumes may have potential for the selection of ecologically adapted and efficient nitrogen-fixing bacteria for soybean inoculation. In this regard, further detailed sampling and analysis of *Bradyrhizobium* populations associated with diverse taxa of native legumes is the topic of a subsequent report.

Different genotypes of soybean (Cregan and Keyser 1986; Lohrke et al. 1996) and *A. bracteata* (Marr et al. 1997) have been reported to vary in their nodulation specificities for specific groups of symbiotic bacteria. Our data based on STRUCTURE analysis suggest that all isolates exhibiting mixed core gene ancestries were sampled exclusively by one of two soybean cultivars. Moreover, there were small, but consistent differences between soybean cultivars used as trap hosts with regard to haplotype, gene, and nucleotide diversity for both core and *nod*C gene sequences of isolates sampled from soybean field sites. Such plant selective effects may reflect the contrasting pedigrees of two soybean cultivars used for bacterial sampling in this study (Voldeng et al. 1996, 1999).

Biogeography and to some extent, legume taxon have been implicated as factors that play a role in structuring *Bradyrhizobium* populations associated with crop legumes (Stepkowski et al. 2005, 2007). In our study, an indication of spatial structuring was provided by the differential recovery of *Bradyrhizobium* lineages at soybean field sites 280 km apart, but differing in soil characteristics, cropping, and inoculation histories. Assuming that random factors (i.e., genetic drift and sampling error) were not responsible for the observed differences, it is tempting to suggest that the exclusive recovery of lineages I and III from one of two sites might reflect regional differences in the distribution of native legume taxa and their associated populations of bradyrhizobia. Support for this proposition was provided by our finding that *A. bracteata* and *D. canadense* were associated with distinct core, and to some extent, symbiotic gene lineages of soybean-nodulating bacteria. Moreover, Spoerke et al. (1996) reported that distinct sub-populations of *A. bracteata* in a natural woodland environment were associated with different lineages of *Bradyrhizobium* defined on the basis of enzyme electrophoresis. Further investigation is obviously needed to elucidate the role of native legume biogeography (at local and regional scales) with regard to the spatial structuring of *Bradyrhizobium* populations associated with crop legumes.

### Microevolution and recombination in bradyrhizobia associated with soybeans

Homologous recombination in bacteria is a major evolutionary force and occurs by the three mechanisms of conjugation, transformation, and transduction (Didelot and Maiden 2010). In this study, use of multiple methods and analyses suggested that homologous recombination had occurred at core loci of soybean-nodulating bacteria from field sites A and B. The rate of recombination relative to mutation (ρ/θ) for the 35 STs from soybean sites, inferred by ClonalFrame reconstruction, indicated that recombination was about 11 times less frequent than mutation. This rate of recombination is significantly lower than an estimate of ρ/θ for three core genes in the symbiotic bacterium, *Rhizobium leguminasarum* (Tian et al. 2010), but is in line with estimates based on core and accessory (virulence) genes in lineages of bacterial pathogens such as *Listeria* (den Bakker et al. 2008) and *Chlamydia* (Joseph et al. 2011). Although our data suggest that mutation was the predominant evolutionary mechanism in soybean-nodulating bacteria, recombination was found to have a significant effect on genetic diversification (r/m **≍**1.0) and was reflected in the direct relationship between nucleotide diversity and total number of inferred recombination events in selected lineages. The effect of recombination on genetic diversification was expected a priori, because recombination, unlike point mutation, affects multiple nucleotides at each event (Didelot et al. 2009). For similar reasons, recombination is considered to represent a much faster mode of evolution than mutation (e.g., den Bakker et al. 2008). This was clearly illustrated by ClonalFrame reconstruction indicating that time to the most recent common ancestor (TMRCA), estimated on the basis of relative branch lengths, was almost three times shorter in the recombination-corrected genealogy of 220 isolates from soybean sites relative to the genealogy without correction. Recombination, particularly when extensive, may have a significant confounding effect on phylogenetic inference by potentially obscuring evolutionary relationships (e.g., Lewis-Rogers et al. 2004). In our study, this effect was evidenced by the extent of phylogenetic uncertainty (reticulation) on inner edges of the “species” network graph of concatenated core gene sequences, and by significant distortion of branch lengths and branching order in the ClonalFrame genealogy without correction for recombination.

Assuming that spatial separation is not a limiting factor, gene flow is generally expected to occur more often between members of the same bacterial species or closely related species than between different species (Didelot and Maiden 2010). On the basis of STRUCTURE analysis, less than 10% of the isolates in five lineages were found to exhibit mixed ancestries, suggesting that interlineage transfer of core genes occurred infrequently. Such infrequent gene flow between lineages is consistent not only with our data indicating substantial divergence and isolation of different evolutionary lineages (based on ClonalFrame reconstruction, population subdivision, and gene flow analyses) but also with novel lineages I, II, and III being considered distinct genomic species. Interestingly, several tests suggested that much of this gene flow was attributable to the *gyr*B locus (coding for type II topoisomerase involved in DNA replication, transcription, recombination, and repair) that was inferred to have an unusual evolutionary history defined by multiple recombination events including interlineage transfers.

Physical proximity is a major requirement for recombination to take place between bacterial members in different lineages (Didelot and Maiden 2010). Pretorius-Guth et al. (1990) suggested that the root nodule may represent the most favorable natural environment for recombination between symbiotic bacteria. However, a recent report (van Berkum et al. 2012) indicated that mixed occupancy of bacterial genotypes in root nodules of field-grown soybeans occurred at a frequency of less than 3% suggesting that mixed infections involving members of different lineages may be an extremely rare event. As the nutrient-rich rhizosphere of the plant host is capable of supporting large bacterial populations, it may provide conditions more suitable for recombination between members of sympatric lineages, particularly in view of evidence for population density-dependant quorum regulation in the bradyrhizobia (e.g., Loh and Stacey 2003).

Differences in the topologies of symbiotic and core gene trees have been attributed to multiple lateral transfers of symbiotic loci among various *Bradyrhizobium* lineages (e.g., Stepkowski et al. 2005, 2007; Steenkamp et al. 2008), whereas other reports (e.g., Moulin et al. 2004; Menna and Hungria 2011) have implicated vertical transmission as the main mechanism of inheritance. In our study, phylogenetic analysis of *nod*C and *nif*H sequences placed all core gene lineages of soybean-nodulating bacteria from field sites A and B, native legumes, and reference taxa in two highly supported symbiotic gene lineages (*nod-nif* I and *nod*-*nif* II) corresponding to *B. japonicum* and *B. elkani*, respectively. In agreement with the conclusions of Moulin et al. (2004) and Menna and Hungria (2011), our data provided several lines of evidence suggesting that vertical transmission was the predominant mechanism of inheritance of symbiotic gene variants in soybean-nodulating bacteria including the inability of multiple tests to detect recombination at either *nod*C or *nif*H loci and the apparent monophyly of the *B. japonicum* clade consisting of core lineages II, III, IV, and V (clustering in the *nod*-*nif* I lineage) in the recombination-corrected ClonalFrame genealogy. Our inferences from ClonalFrame reconstruction are also consistent with a proposal by Itakura et al. (2009) that an ancestor of “*B. japonicum*” (*B. japonicum* clade in this study) may have diverged into different lineages after the acquisition of a symbiosis island region (genomic island).

In conclusion, our study has not only provided new insights into the role of homologous recombination in the genetic diversification of lineages of soybean-nodulating bacteria but also strongly suggests that soybeans in east North America are predominantly colonized by bradyrhizobia originating from native legumes, irrespective of the practice of recurrent inoculation. Moreover, our study highlights the potential of sequence-based inference methods for detailed studies on the microevolution and ecology of populations of symbiotic bacteria. The sequencing of more genes (up to whole genomes) may provide greater insight into the microevolutionary dynamics of these populations.

## Supporting Information

**Table S1.** Location of housekeeping and symbiotic genes used in this study on the chromosomes of *Bradyrhizobium japonicum* USDA6T (GenBank, NC017249) and USDA110 (GenBank, NC004463).

**Table S2.** GenBank accession numbers of housekeeping (*atp*D, *gln*II, *rec*A, *gyr*B, *rpo*B, *dna*K) and symbiotic (*nod*C) partial gene sequences representing 220 *Bradyrhizobium* isolates from soybean field sites A and B.

**Table S3.** GenBank nucleotide sequence accession numbers for *Bradyrhizobium* reference strains.

**Table S4.** Nucleotide accession numbers of 148 partial *rec*A gene sequences representing soybean-nodulating bacteria from *Amphicarpaea bracteata* and *Desmodium canadense*.

**Table S5.** GenBank nucleotide accession numbers for (a) selected soybean-nodulating bacteria from *Amphicarpaea bracteata* and *Desmodium canadense* (*dna*K, *nod*C, and *nif*H partial gene sequences) and (b) reference strains and selected isolates from soybean field sites A and B (*nif*H partial gene sequences).

**Table S6.** Primers and DNA amplification conditions.

**Table S7.** Example of R scripts based on the phangorn phylogenetic package to test the extent of congruence between maximum-likelihood trees reconstructed for each of six housekeeping genes.

**Table S8.** Statistics for five separate runs of STRUCTURE using the admixture model (independent allele frequencies) for values of K between 2 and 7.

**Table S9.** Genetic diversity statistics for *Bradyrhizobium* isolates from soybean cultivars M and O.

**Table S10.** Genetic differentiation and gene flow statistics for selected core gene lineages based on six concatenated gene sequences.

**Figure S1.** Maximum-likelihood tree of concatenated *atp*D-*gln*II-*rec*A-*gyr*B-*rpo*B-*dna*K partial gene sequences (3210 bp) for *Bradyrhizobium* reference strains and 35 unique STs representing soybean-nodulating bacteria from field sites A and B.

**Figure S2.** Randomization test to assess congruence between maximum-likelihood (ML) trees of six housekeeping genes and 100 random trees.

**Figure S3.** Distribution of external to internal branch length ratios of a tree of lineage II (*n* = 120) inferred by ClonalFrame relative to that expected under coalescent simulation.

**Figure S4.** Maximum-likelihood tree of unique *gyr*B partial gene sequences (618 bp) of *Bradyrhizobium* isolates from soybean field sites A and B and reference taxa showing putative xenologous sequences.

**Figure S5.** Maximum-likelihood phylogenetic trees of (a) *rec*A (462 bp) and (b) concatenated *rec*A-*dna*K (831 bp) partial gene sequences representing soybean-nodulating bacteria from *Amphicarpaea bracteata* and *Desmodium canadense*, from soybean field sites A and B, and, reference taxa.
