# Supplementary material for: Microevolution of symbiotic Bradyrhizobium populations associated with soybeans in east North America
Source: Ecol Evol. 2012 Oct 22;2(12):2943–61. doi: 10.1002/ece3.404 (PMC3538991; doi:10.1002/ece3.404)

Supporting Information

Table S1 Location of housekeeping and symbiotic genes used in this study on the chromosomes of *Bradyrhizobium japonicum* USDA6^T (GenBank, NC017249) and USDA110 (GenBank, NC004463).

The chromosomal location of putative symbiosis islands are indicated.

Gene	USDA6^T chromosome (9.207 Mb)			USDA110 chromosome (9.106 Mb)		
	Start position (Mb)	End position (Mb)	Locus tag	Start position (Mb)	End position (Mb)	Locus tag
			Housekeeping genes			
*atp*D	0.423	0.424	BJ6T04050	0.474	0.475	bll0440
*dna*K	6.752	6.753	BJ6T66070	0.725	0.727	blr0678
*gln*II	5.702	5.703	BJ6T56300	4.607	4.608	blr4169
*gyr*B	0.873	0.875	BJ6T08290	0.884	0.886	bll0823
*rec*A	4.103	4.104	BJ6T39800	6.326	6.326	bll5755
*rpo*B	4.472	4.476	BJ6T43770	5.952	5.956	bll5410
			Symbiotic genes			
*nif*H	8.325	8.326	BJ6T80500	1.929	1.929	blr1769
*nod*C	8.091	8.092	BJ6T78270	2.185	2.187	blr2027
Location of putative symbiosis islands						
Island A	7.920	8.614	-	1.681	2.362	-
Island B	2.248	2.253	-	7.934	7.940	-
Island C	9.114	0 - 0.070	-	8.98	0 - 0.08	-

Table S2 GenBank accession numbers of housekeeping (*atp*D, *gln*II, *rec*A, *gyr*B, *rpo*B, *dna*K) and symbiotic (*nod*C) partial gene sequences representing 220 *Bradyrhizobium* isolates from soybean field sites A and B.

All sequences were generated in this study. Bacterial isolates are designated as follows: the first letter represents field site of origin (O, Ontario; H, Quebec), the second letter represents the soybean cultivar used for isolation (O, AC Orford; M, Maple Glen); numbers after letters refer to isolate numbers. ST designates the sequence type number.

Isolate	ST	*atpD*	*glnII*	*recA*	*gyrB*	*rpoB*	*dnaK*	*nodC*
OM1	15	HQ455114	HQ587652	HQ587189	HQ873081	HQ587420	JF308718	HQ587882
OM2	21	HQ455115	HQ587653	HQ587190	HQ873082	HQ587421	JF308719	HQ587883
OM3	21	HQ455116	HQ587654	HQ587191	HQ873083	HQ587422	JF308720	HQ587884
OM4	21	HQ455117	HQ587655	HQ587192	HQ873084	HQ587423	JF308721	HQ587885
OM5	21	HQ455118	HQ587656	HQ587193	HQ873085	HQ587424	JF308722	HQ587886
OM6	34	HQ455119	HQ587657	HQ587194	HQ873086	HQ587425	JF308723	HQ587887
OM7	21	HQ455120	HQ587658	HQ587195	HQ873087	HQ587426	JF308724	HQ587888
OM8	15	HQ455121	HQ587659	HQ587196	HQ873088	HQ587427	JF308725	HQ587889
OM9	34	HQ455122	HQ587660	HQ587197	HQ873089	HQ587428	JF308726	HQ587890
OM10	15	HQ455123	HQ587661	HQ587198	HQ873090	HQ587429	JF308727	HQ587891
OM11	21	HQ455124	HQ587662	HQ587199	HQ873091	HQ587430	JF308728	HQ587892
OM12	15	HQ455125	HQ587663	HQ587200	HQ873092	HQ587431	JF308729	HQ587893
OM13	15	HQ455126	HQ587664	HQ587201	HQ873093	HQ587432	JF308730	HQ587894
OM14	20	HQ455127	HQ587665	HQ587202	HQ873094	HQ587433	JF308731	HQ587895
OM15	11	HQ455128	HQ587666	HQ587203	HQ873095	HQ587434	JF308732	HQ587896
OM16	21	HQ455129	HQ587667	HQ587204	HQ873096	HQ587435	JF308733	HQ587897
OM17	7	HQ455130	HQ587668	HQ587205	HQ873097	HQ587436	JF308734	HQ587898
OM18	15	HQ455131	HQ587669	HQ587206	HQ873098	HQ587437	JF308735	HQ587899
OM19	21	HQ455132	HQ587670	HQ587207	HQ873099	HQ587438	JF308736	HQ587900
OM20	18	HQ455133	HQ587671	HQ587208	HQ873100	HQ587439	JF308737	HQ587901
OM21	11	HQ455134	HQ587672	HQ587209	HQ873101	HQ587440	JF308738	HQ587902
OM22	21	HQ455135	HQ587673	HQ587210	HQ873102	HQ587441	JF308739	HQ587903
OM23	21	HQ455136	HQ587674	HQ587211	HQ873103	HQ587442	JF308740	HQ587904
OM24	11	HQ455137	HQ587675	HQ587212	HQ873104	HQ587443	JF308741	HQ587905
OM25	21	HQ455138	HQ587676	HQ587213	HQ873105	HQ587444	JF308742	HQ587906
OM26	27	HQ455139	HQ587677	HQ587214	HQ873106	HQ587445	JF308743	HQ587907
OM27	34	HQ455140	HQ587678	HQ587215	HQ873107	HQ587446	JF308744	HQ587908
OM28	28	HQ455141	HQ587679	HQ587216	HQ873108	HQ587447	JF308745	HQ587909
OM29	21	HQ455142	HQ587680	HQ587217	HQ873109	HQ587448	JF308746	HQ587910
OM30	20	HQ455143	HQ587681	HQ587218	HQ873110	HQ587449	JF308747	HQ587911
OM31	7	HQ455144	HQ587682	HQ587219	HQ873111	HQ587450	JF308748	HQ587912
OM32	21	HQ455145	HQ587683	HQ587220	HQ873112	HQ587451	JF308749	HQ587913

OM33	11	HQ455146	HQ587684	HQ587221	HQ873113	HQ587452	JF308750	HQ587914
OM34	15	HQ455147	HQ587685	HQ587222	HQ873114	HQ587453	JF308751	HQ587915
OM35	21	HQ455148	HQ587686	HQ587223	HQ873115	HQ587454	JF308752	HQ587916
OM36	20	HQ455149	HQ587687	HQ587224	HQ873116	HQ587455	JF308753	HQ587917
OM37	15	HQ455150	HQ587688	HQ587225	HQ873117	HQ587456	JF308754	HQ587918
OM38	21	HQ455151	HQ587689	HQ587226	HQ873118	HQ587457	JF308755	HQ587919
OM39	15	HQ455152	HQ587690	HQ587227	HQ873119	HQ587458	JF308756	HQ587920
OM40	11	HQ455153	HQ587691	HQ587228	HQ873120	HQ587459	JF308757	HQ587921
OM41	13	HQ455154	HQ587692	HQ587229	HQ873121	HQ587460	JF308758	HQ587922
OM42	21	HQ455155	HQ587693	HQ587230	HQ873122	HQ587461	JF308759	HQ587923
OM43	15	HQ455156	HQ587694	HQ587231	HQ873123	HQ587462	JF308760	HQ587924
OM44	21	HQ455157	HQ587695	HQ587232	HQ873124	HQ587463	JF308761	HQ587925
OM45	15	HQ455158	HQ587696	HQ587233	HQ873125	HQ587464	JF308762	HQ587926
OM46	15	HQ455159	HQ587697	HQ587234	HQ873126	HQ587465	JF308763	HQ587927
OM47	15	HQ455160	HQ587698	HQ587235	HQ873127	HQ587466	JF308764	HQ587928
OM48	21	HQ455161	HQ587699	HQ587236	HQ873128	HQ587467	JF308765	HQ587929
OM49	21	HQ455162	HQ587700	HQ587237	HQ873129	HQ587468	JF308766	HQ587930
OM50	4	HQ455163	HQ587701	HQ587238	HQ873130	HQ587469	JF308767	HQ587931
OM51	21	HQ455164	HQ587702	HQ587239	HQ873131	HQ587470	JF308768	HQ587932
OM52	21	HQ455165	HQ587703	HQ587240	HQ873132	HQ587471	JF308769	HQ587933
OM53	21	HQ455166	HQ587704	HQ587241	HQ873133	HQ587472	JF308770	HQ587934
OM54	21	HQ455167	HQ587705	HQ587242	HQ873134	HQ587473	JF308771	HQ587935
OM55	21	HQ455168	HQ587706	HQ587243	HQ873135	HQ587474	JF308772	HQ587936
OO56	29	HQ455169	HQ587707	HQ587244	HQ873136	HQ587475	JF308773	HQ587937
OO57	34	HQ455170	HQ587708	HQ587245	HQ873137	HQ587476	JF308774	HQ587938
OO58	21	HQ455171	HQ587709	HQ587246	HQ873138	HQ587477	JF308775	HQ587939
OO59	21	HQ455172	HQ587710	HQ587247	HQ873139	HQ587478	JF308776	HQ587940
OO60	33	HQ455173	HQ587711	HQ587248	HQ873140	HQ587479	JF308777	HQ587941
OO61	27	HQ455174	HQ587712	HQ587249	HQ873141	HQ587480	JF308778	HQ587942
OO62	21	HQ455175	HQ587713	HQ587250	HQ873142	HQ587481	JF308779	HQ587943
OO63	15	HQ455176	HQ587714	HQ587251	HQ873143	HQ587482	JF308780	HQ587944
OO64	21	HQ455177	HQ587715	HQ587252	HQ873144	HQ587483	JF308781	HQ587945
OO65	15	HQ455178	HQ587716	HQ587253	HQ873145	HQ587484	JF308782	HQ587946
OO66	34	HQ455179	HQ587717	HQ587254	HQ873146	HQ587485	JF308783	HQ587947
OO67	15	HQ455180	HQ587718	HQ587255	HQ873147	HQ587486	JF308784	HQ587948
OO68	15	HQ455181	HQ587719	HQ587256	HQ873148	HQ587487	JF308785	HQ587949
OO69	21	HQ455182	HQ587720	HQ587257	HQ873149	HQ587488	JF308786	HQ587950
OO70	21	HQ455183	HQ587721	HQ587258	HQ873150	HQ587489	JF308787	HQ587951
OO71	15	HQ455184	HQ587722	HQ587259	HQ873151	HQ587490	JF308788	HQ587952
OO72	30	HQ455185	HQ587723	HQ587260	HQ873152	HQ587491	JF308789	HQ587953
OO73	15	HQ455186	HQ587724	HQ587261	HQ873153	HQ587492	JF308790	HQ587954
OO74	33	HQ455187	HQ587725	HQ587262	HQ873154	HQ587493	JF308791	HQ587955
OO75	21	HQ455188	HQ587726	HQ587263	HQ873155	HQ587494	JF308792	HQ587956
OO76	21	HQ455189	HQ587727	HQ587264	HQ873156	HQ587495	JF308793	HQ587957
OO77	33	HQ455190	HQ587728	HQ587265	HQ873157	HQ587496	JF308794	HQ587958

OO78	15	HQ455191	HQ587729	HQ587266	HQ873158	HQ587497	JF308795	HQ587959
OO79	30	HQ455192	HQ587730	HQ587267	HQ873159	HQ587498	JF308796	HQ587960
OO80	27	HQ455193	HQ587731	HQ587268	HQ873160	HQ587499	JF308797	HQ587961
OO81	33	HQ455194	HQ587732	HQ587269	HQ873161	HQ587500	JF308798	HQ587962
OO82	27	HQ455195	HQ587733	HQ587270	HQ873162	HQ587501	JF308799	HQ587963
OO83	15	HQ455196	HQ587734	HQ587271	HQ873163	HQ587502	JF308800	HQ587964
OO84	33	HQ455197	HQ587735	HQ587272	HQ873164	HQ587503	JF308801	HQ587965
OO85	33	HQ455198	HQ587736	HQ587273	HQ873165	HQ587504	JF308802	HQ587966
OO86	34	HQ455199	HQ587737	HQ587274	HQ873166	HQ587505	JF308803	HQ587967
OO87	27	HQ455200	HQ587738	HQ587275	HQ873167	HQ587506	JF308804	HQ587968
OO88	21	HQ455201	HQ587739	HQ587276	HQ873168	HQ587507	JF308805	HQ587969
OO89	21	HQ455202	HQ587740	HQ587277	HQ873169	HQ587508	JF308806	HQ587970
OO90	21	HQ455203	HQ587741	HQ587278	HQ873170	HQ587509	JF308807	HQ587971
OO91	21	HQ455204	HQ587742	HQ587279	HQ873171	HQ587510	JF308808	HQ587972
OO92	15	HQ455205	HQ587743	HQ587280	HQ873172	HQ587511	JF308809	HQ587973
OO93	27	HQ455206	HQ587744	HQ587281	HQ873173	HQ587512	JF308810	HQ587974
OO94	34	HQ455207	HQ587745	HQ587282	HQ873174	HQ587513	JF308811	HQ587975
OO95	21	HQ455208	HQ587746	HQ587283	HQ873175	HQ587514	JF308812	HQ587976
OO96	27	HQ455209	HQ587747	HQ587284	HQ873176	HQ587515	JF308813	HQ587977
OO97	21	HQ455210	HQ587748	HQ587285	HQ873177	HQ587516	JF308814	HQ587978
OO98	21	HQ455211	HQ587749	HQ587286	HQ873178	HQ587517	JF308815	HQ587979
OO99	35	HQ455212	HQ587750	HQ587287	HQ873179	HQ587518	JF308816	HQ587980
OO100	34	HQ455213	HQ587751	HQ587288	HQ873180	HQ587519	JF308817	HQ587981
OO101	33	HQ455214	HQ587752	HQ587289	HQ873181	HQ587520	JF308818	HQ587982
OO102	21	HQ455215	HQ587753	HQ587290	HQ873182	HQ587521	JF308819	HQ587983
OO103	15	HQ455216	HQ587754	HQ587291	HQ873183	HQ587522	JF308820	HQ587984
OO104	27	HQ455217	HQ587755	HQ587292	HQ873184	HQ587523	JF308821	HQ587985
OO105	21	HQ455218	HQ587756	HQ587293	HQ873185	HQ587524	JF308822	HQ587986
OO106	15	HQ455219	HQ587757	HQ587294	HQ873186	HQ587525	JF308823	HQ587987
OO107	15	HQ455220	HQ587758	HQ587295	HQ873187	HQ587526	JF308824	HQ587988
OO108	15	HQ455221	HQ587759	HQ587296	HQ873188	HQ587527	JF308825	HQ587989
OO109	15	HQ455222	HQ587760	HQ587297	HQ873189	HQ587528	JF308826	HQ587990
OO110	15	HQ455223	HQ587761	HQ587298	HQ873190	HQ587529	JF308827	HQ587991
HM111	14	HQ455224	HQ587762	HQ587299	HQ873191	HQ587530	JF308828	HQ587992
HM112	21	HQ455225	HQ587763	HQ587300	HQ873192	HQ587531	JF308829	HQ587993
HM113	15	HQ455226	HQ587764	HQ587301	HQ873193	HQ587532	JF308830	HQ587994
HM114	14	HQ455227	HQ587765	HQ587302	HQ873194	HQ587533	JF308831	HQ587995
HM115	14	HQ455228	HQ587766	HQ587303	HQ873195	HQ587534	JF308832	HQ587996
HM116	32	HQ455229	HQ587767	HQ587304	HQ873196	HQ587535	JF308833	HQ587997
HM117	21	HQ455230	HQ587768	HQ587305	HQ873197	HQ587536	JF308834	HQ587998
HM118	21	HQ455231	HQ587769	HQ587306	HQ873198	HQ587537	JF308835	HQ587999
HM119	3	HQ455232	HQ587770	HQ587307	HQ873199	HQ587538	JF308836	HQ588000
HM120	15	HQ455233	HQ587771	HQ587308	HQ873200	HQ587539	JF308837	HQ588001
HM121	8	HQ455234	HQ587772	HQ587309	HQ873201	HQ587540	JF308838	HQ588002
HM122	3	HQ455235	HQ587773	HQ587310	HQ873202	HQ587541	JF308839	HQ588003

HM123	21	HQ455236	HQ587774	HQ587311	HQ873203	HQ587542	JF308840	HQ588004
HM124	14	HQ455237	HQ587775	HQ587312	HQ873204	HQ587543	JF308841	HQ588005
HM125	15	HQ455238	HQ587776	HQ587313	HQ873205	HQ587544	JF308842	HQ588006
HM126	3	HQ455239	HQ587777	HQ587314	HQ873206	HQ587545	JF308843	HQ588007
HM127	13	HQ455240	HQ587778	HQ587315	HQ873207	HQ587546	JF308844	HQ588008
HM128	8	HQ455241	HQ587779	HQ587316	HQ873208	HQ587547	JF308845	HQ588009
HM129	14	HQ455242	HQ587780	HQ587317	HQ873209	HQ587548	JF308846	HQ588010
HM130	13	HQ455243	HQ587781	HQ587318	HQ873210	HQ587549	JF308847	HQ588011
HM131	32	HQ455244	HQ587782	HQ587319	HQ873211	HQ587550	JF308848	HQ588012
HM132	14	HQ455245	HQ587783	HQ587320	HQ873212	HQ587551	JF308849	HQ588013
HM133	21	HQ455246	HQ587784	HQ587321	HQ873213	HQ587552	JF308850	HQ588014
HM134	14	HQ455247	HQ587785	HQ587322	HQ873214	HQ587553	JF308851	HQ588015
HM135	21	HQ455248	HQ587786	HQ587323	HQ873215	HQ587554	JF308852	HQ588016
HM136	14	HQ455249	HQ587787	HQ587324	HQ873216	HQ587555	JF308853	HQ588017
HM137	21	HQ455250	HQ587788	HQ587325	HQ873217	HQ587556	JF308854	HQ588018
HM138	14	HQ455251	HQ587789	HQ587326	HQ873218	HQ587557	JF308855	HQ588019
HM139	3	HQ455252	HQ587790	HQ587327	HQ873219	HQ587558	JF308856	HQ588020
HM140	32	HQ455253	HQ587791	HQ587328	HQ873220	HQ587559	JF308857	HQ588021
HM141	14	HQ455254	HQ587792	HQ587329	HQ873221	HQ587560	JF308858	HQ588022
HM142	21	HQ455255	HQ587793	HQ587330	HQ873222	HQ587561	JF308859	HQ588023
HM143	21	HQ455256	HQ587794	HQ587331	HQ873223	HQ587562	JF308860	HQ588024
HM144	7	HQ455257	HQ587795	HQ587332	HQ873224	HQ587563	JF308861	HQ588025
HM145	15	HQ455258	HQ587796	HQ587333	HQ873225	HQ587564	JF308862	HQ588026
HM146	8	HQ455259	HQ587797	HQ587334	HQ873226	HQ587565	JF308863	HQ588027
HM147	7	HQ455260	HQ587798	HQ587335	HQ873227	HQ587566	JF308864	HQ588028
HM148	14	HQ455261	HQ587799	HQ587336	HQ873228	HQ587567	JF308865	HQ588029
HM149	14	HQ455262	HQ587800	HQ587337	HQ873229	HQ587568	JF308866	HQ588030
HM150	3	HQ455263	HQ587801	HQ587338	HQ873230	HQ587569	JF308867	HQ588031
HM151	8	HQ455264	HQ587802	HQ587339	HQ873231	HQ587570	JF308868	HQ588032
HM152	21	HQ455265	HQ587803	HQ587340	HQ873232	HQ587571	JF308869	HQ588033
HM153	23	HQ455266	HQ587804	HQ587341	HQ873233	HQ587572	JF308870	HQ588034
HM154	14	HQ455267	HQ587805	HQ587342	HQ873234	HQ587573	JF308871	HQ588035
HM155	9	HQ455268	HQ587806	HQ587343	HQ873235	HQ587574	JF308872	HQ588036
HM156	3	HQ455269	HQ587807	HQ587344	HQ873236	HQ587575	JF308873	HQ588037
HM157	3	HQ455270	HQ587808	HQ587345	HQ873237	HQ587576	JF308874	HQ588038
HM158	3	HQ455271	HQ587809	HQ587346	HQ873238	HQ587577	JF308875	HQ588039
HM159	21	HQ455272	HQ587810	HQ587347	HQ873239	HQ587578	JF308876	HQ588040
HM160	3	HQ455273	HQ587811	HQ587348	HQ873240	HQ587579	JF308877	HQ588041
HM161	14	HQ455274	HQ587812	HQ587349	HQ873241	HQ587580	JF308878	HQ588042
HM162	14	HQ455275	HQ587813	HQ587350	HQ873242	HQ587581	JF308879	HQ588043
HM163	15	HQ455276	HQ587814	HQ587351	HQ873243	HQ587582	JF308880	HQ588044
HM164	14	HQ455277	HQ587815	HQ587352	HQ873244	HQ587583	JF308881	HQ588045
HM165	3	HQ455278	HQ587816	HQ587353	HQ873245	HQ587584	JF308882	HQ588046
HO166	11	HQ455279	HQ587817	HQ587354	HQ873246	HQ587585	JF308883	HQ588047
HO167	3	HQ455280	HQ587818	HQ587355	HQ873247	HQ587586	JF308884	HQ588048

HO168	32	HQ455281	HQ587819	HQ587356	HQ873248	HQ587587	JF308885	HQ588049
HO169	12	HQ455282	HQ587820	HQ587357	HQ873249	HQ587588	JF308886	HQ588050
HO170	21	HQ455283	HQ587821	HQ587358	HQ873250	HQ587589	JF308887	HQ588051
HO171	6	HQ455284	HQ587822	HQ587359	HQ873251	HQ587590	JF308888	HQ588052
HO172	25	HQ455285	HQ587823	HQ587360	HQ873252	HQ587591	JF308889	HQ588053
HO173	6	HQ455286	HQ587824	HQ587361	HQ873253	HQ587592	JF308890	HQ588054
HO174	1	HQ455287	HQ587825	HQ587362	HQ873254	HQ587593	JF308891	HQ588055
HO175	32	HQ455288	HQ587826	HQ587363	HQ873255	HQ587594	JF308892	HQ588056
HO176	15	HQ455289	HQ587827	HQ587364	HQ873256	HQ587595	JF308893	HQ588057
HO177	22	HQ455290	HQ587828	HQ587365	HQ873257	HQ587596	JF308894	HQ588058
HO178	17	HQ455291	HQ587829	HQ587366	HQ873258	HQ587597	JF308895	HQ588059
HO179	22	HQ455292	HQ587830	HQ587367	HQ873259	HQ587598	JF308896	HQ588060
HO180	10	HQ455293	HQ587831	HQ587368	HQ873260	HQ587599	JF308897	HQ588061
HO181	1	HQ455294	HQ587832	HQ587369	HQ873261	HQ587600	JF308898	HQ588062
HO182	31	HQ455295	HQ587833	HQ587370	HQ873262	HQ587601	JF308899	HQ588063
HO183	26	HQ455296	HQ587834	HQ587371	HQ873263	HQ587602	JF308900	HQ588064
HO184	16	HQ455297	HQ587835	HQ587372	HQ873264	HQ587603	JF308901	HQ588065
HO185	3	HQ455298	HQ587836	HQ587373	HQ873265	HQ587604	JF308902	HQ588066
HO186	31	HQ455299	HQ587837	HQ587374	HQ873266	HQ587605	JF308903	HQ588067
HO187	11	HQ455300	HQ587838	HQ587375	HQ873267	HQ587606	JF308904	HQ588068
HO188	27	HQ455301	HQ587839	HQ587376	HQ873268	HQ587607	JF308905	HQ588069
HO189	15	HQ455302	HQ587840	HQ587377	HQ873269	HQ587608	JF308906	HQ588070
HO190	2	HQ455303	HQ587841	HQ587378	HQ873270	HQ587609	JF308907	HQ588071
HO191	19	HQ455304	HQ587842	HQ587379	HQ873271	HQ587610	JF308908	HQ588072
HO192	5	HQ455305	HQ587843	HQ587380	HQ873272	HQ587611	JF308909	HQ588073
HO193	3	HQ455306	HQ587844	HQ587381	HQ873273	HQ587612	JF308910	HQ588074
HO194	15	HQ455307	HQ587845	HQ587382	HQ873274	HQ587613	JF308911	HQ588075
HO195	3	HQ455308	HQ587846	HQ587383	HQ873275	HQ587614	JF308912	HQ588076
HO196	24	HQ455309	HQ587847	HQ587384	HQ873276	HQ587615	JF308913	HQ588077
HO197	3	HQ455310	HQ587848	HQ587385	HQ873277	HQ587616	JF308914	HQ588078
HO198	32	HQ455311	HQ587849	HQ587386	HQ873278	HQ587617	JF308915	HQ588079
HO199	32	HQ455312	HQ587850	HQ587387	HQ873279	HQ587618	JF308916	HQ588080
HO200	22	HQ455313	HQ587851	HQ587388	HQ873280	HQ587619	JF308917	HQ588081
HO201	3	HQ455314	HQ587852	HQ587389	HQ873281	HQ587620	JF308918	HQ588082
HO202	9	HQ455315	HQ587853	HQ587390	HQ873282	HQ587621	JF308919	HQ588083
HO203	3	HQ455316	HQ587854	HQ587391	HQ873283	HQ587622	JF308920	HQ588084
HO204	3	HQ455317	HQ587855	HQ587392	HQ873284	HQ587623	JF308921	HQ588085
HO205	32	HQ455318	HQ587856	HQ587393	HQ873285	HQ587624	JF308922	HQ588086
HO206	11	HQ455319	HQ587857	HQ587394	HQ873286	HQ587625	JF308923	HQ588087
HO207	22	HQ455320	HQ587858	HQ587395	HQ873287	HQ587626	JF308924	HQ588088
HO208	3	HQ455321	HQ587859	HQ587396	HQ873288	HQ587627	JF308925	HQ588089
HO209	3	HQ455322	HQ587860	HQ587397	HQ873289	HQ587628	JF308926	HQ588090
HO210	7	HQ455323	HQ587861	HQ587398	HQ873290	HQ587629	JF308927	HQ588091
HO211	11	HQ455324	HQ587862	HQ587399	HQ873291	HQ587630	JF308928	HQ588092
HO212	28	HQ455325	HQ587863	HQ587400	HQ873292	HQ587631	JF308929	HQ588093

HO213	22	HQ455326	HQ587864	HQ587401	HQ873293	HQ587632	JF308930	HQ588094
HO214	22	HQ455327	HQ587865	HQ587402	HQ873294	HQ587633	JF308931	HQ588095
HO215	32	HQ455328	HQ587866	HQ587403	HQ873295	HQ587634	JF308932	HQ588096
HO216	22	HQ455329	HQ587867	HQ587404	HQ873296	HQ587635	JF308933	HQ588097
HO217	28	HQ455330	HQ587868	HQ587405	HQ873297	HQ587636	JF308934	HQ588098
HO218	7	HQ455331	HQ587869	HQ587406	HQ873298	HQ587637	JF308935	HQ588099
HO219	32	HQ455332	HQ587870	HQ587407	HQ873299	HQ587638	JF308936	HQ588100
HO220	3	HQ455333	HQ587871	HQ587408	HQ873300	HQ587639	JF308937	HQ588101

Table S3 GenBank nucleotide sequence accession numbers for *Bradyrhizobium* reference strains.

Sequences in bold were generated in this study. Asterisks denote strains (*B. betae* LMG21987^{T}and *Bradyrhizobium* sp. BTAi1) that do not possess the *nod*C gene.

Reference strain	*atpD*	*glnII*	*recA*	*gyrB*	*rpoB*	*dnaK*	*nodC*
B. betae LMG21987^T	FM253129	AB353733	AB353734	FM253217	FM253260	AY923046	*
B. canariense LMG22265^T	FM253135	AY386765	AY591553	FM253220	FM253263	AY923047	AJ560653
B. cytisi LMG25866^T	**JN186289**	**JN186291**	**JN186293**	**JN186292**	**JN186288**	**JN186290**	EU597844
B. elkanii USDA76^T	AY386758	AY599117	AM591568	AM418800	EF190188	AY328392	AB354631
B. iriomotense LMG24129^T	AB300994	AB300995	AB300996	**HQ873308**	**HQ587646**	**JF308944**	-
B. japonicum USDA6^T	AM168320	**HQ587875**	AM168341	AM418801	AY242830	AM168362	AB354632
B. jicamae LMG24556^T	FJ428211	FJ428204	**HQ587415**	**HQ873309**	**HQ587647**	**JF308945**	**HQ588109**
B. liaoningense LMG18230^T	FM253137	AY386775	AY591564	FM253223	FM253266	AY923041	-
B. pachyrhizi LMG24246^T	FJ428208	FJ428201	**HQ587416**	**HQ873310**	**HQ587648**	**JF308946**	**HQ588110**
B. yuanmingense LMG21827^T	FM253140	AY386780	AM168343	FM253226	FM253269	AY923039	AB354633
Bradyrhizobium sp. 532C	**HQ455336**	**HQ587874**	**HQ587411**	**HQ873303**	**HQ587642**	**JF308940**	**HQ588104**
Bradyrhizobium sp. USDA 110	BA000040	BA000040	BA000040	BA000040	BA000040	BA000040	BA000040
Bradyrhizobium sp. USDA 122	AY653767	AY386777	AY591562	**HQ873304**	EF190186	AY923044	**HQ588105**
Bradyrhizobium sp. USDA 136	**HQ455337**	**HQ587876**	**HQ587412**	**HQ873305**	**HQ587643**	**JF308941**	**HQ588106**
Bradyrhizobium sp. USDA 138	**HQ455338**	**HQ587877**	**HQ587413**	**HQ873306**	**HQ587644**	**JF308942**	**HQ588107**
Bradyrhizobium sp. USDA 142	**HQ455339**	**HQ587878**	**HQ587414**	**HQ873307**	**HQ587645**	**JF308943**	**HQ588108**
Bradyrhizobium sp. 61A101	**HQ455334**	**HQ587872**	**HQ587409**	**HQ873301**	**HQ587640**	**JF308938**	**HQ588102**
Bradyrhizobium sp. 61A124	**HQ455335**	**HQ587873**	**HQ587410**	**HQ873302**	**HQ587641**	**JF308939**	**HQ588103**
Bradyrhizobium sp. BTAi1	CP000494	CP000494	CP000494	CP000494	CP000494	CP000494	*

Table S4 Nucleotide accession numbers of 148 partial *rec*A gene sequences representing soybean-nodulating bacteria from *A. bracteata* and *D. canadense*.

All sequences were generated in this study. Isolate numbers designated S2 and S4 are from *D. canadense* (Aylmer, Quebec) and *A. bracteata* (Donnacona, Quebec), respectively. Lineage numbers inferred by maximum-likelihood analyses are shown in parentheses.

Isolate No. (Lineage)	Accession No.	Isolate No. (Lineage)	Accession No.	Isolate No. (Lineage)	Accession No.	Isolate No. (Lineage)	Accession No.
176S2 (V)	JQ783158	275S2 (V)	JQ783195	315S2 (V)	JQ783232	359S2 (V)	JQ783269
177S2 (V)	JQ783159	276S2 (V)	JQ783196	316S2 (V)	JQ783233	360S2 (V)	JQ783270
178S2 (V)	JQ783160	277S2 (V)	JQ783197	317S2 (V)	JQ783234	362S2 (V)	JQ783271
180S2 (V)	JQ783161	278S2 (V)	JQ783198	319S2 (V)	JQ783235	363S2 (V)	JQ783272
181S2 (V)	JQ783162	279S2 (V)	JQ783199	320S2 (V)	JQ783236	364S2 (V)	JQ783273
237S2 (V)	JQ783163	280S2 (V)	JQ783200	321S2 (V)	JQ783237	365S2 (V)	JQ783274
238S2 (V)	JQ783164	281S2 (V)	JQ783201	322S2 (V)	JQ783238	366S2 (V)	JQ783275
239S2 (V)	JQ783165	282S2 (V)	JQ783202	323S2 (V)	JQ783239	367S2 (V)	JQ783276
240S2 (V)	JQ783166	284S2 (V)	JQ783203	324S2 (V)	JQ783240	1S4 (II)	JQ783305
241S2 (V)	JQ783167	285S2 (V)	JQ783204	325S2 (V)	JQ783241	2S4 (I)	JQ783306
244S2 (V)	JQ783168	286S2 (V)	JQ783205	326S2 (V)	JQ783242	3S4 (I)	JQ783307
245S2 (V)	JQ783169	287S2 (V)	JQ783206	327S2 (V)	JQ783243	4S4 (II)	JQ783308
246S2 (V)	JQ783170	288S2 (V)	JQ783207	328S2 (V)	JQ783244	5S4 (I)	JQ783309
247S2 (V)	JQ783171	289S2 (V)	JQ783208	329S2 (V)	JQ783245	6S4 (II)	JQ783310
248S2 (V)	JQ783172	290S2 (V)	JQ783209	330S2 (V)	JQ783246	7S4 (I)	JQ783311
250S2 (V)	JQ783173	291S2 (V)	JQ783210	331S2 (V)	JQ783247	8S4 (II)	JQ783312
252S2 (V)	JQ783174	292S2 (V)	JQ783211	332S2 (V)	JQ783248	12S4 (I)	JQ783313
253S2 (V)	JQ783175	293S2 (V)	JQ783212	334S2 (V)	JQ783249	13S4 (II)	JQ783314
254S2 (V)	JQ783176	294S2 (V)	JQ783213	335S2 (V)	JQ783250	14S4 (II)	JQ783315
255S2 (V)	JQ783177	295S2 (V)	JQ783214	336S2 (V)	JQ783251	15S4 (II)	JQ783316
256S2 (V)	JQ783178	296S2 (V)	JQ783215	337S2 (V)	JQ783252	18S4 (II)	JQ783317
257S2 (V)	JQ783179	297S2 (V)	JQ783216	338S2 (V)	JQ783253	19S4 (II)	JQ783318
258S2 (V)	JQ783180	298S2 (V)	JQ783217	341S2 (V)	JQ783254	20S4 (II)	JQ783319
259S2 (V)	JQ783181	299S2 (V)	JQ783218	342S2 (V)	JQ783255	21S4 (II)	JQ783320
260S2 (V)	JQ783182	300S2 (V)	JQ783219	343S2 (V)	JQ783256	22S4 (II)	JQ783321
261S2 (V)	JQ783183	301S2 (V)	JQ783220	344S2 (V)	JQ783257	23S4 (I)	JQ783322
262S2 (V)	JQ783184	302S2 (V)	JQ783221	345S2 (V)	JQ783258	24S4 (II)	JQ783323
263S2 (V)	JQ783185	303S2 (V)	JQ783222	346S2 (V)	JQ783259	25S4 (II)	JQ783324
264S2 (V)	JQ783186	304S2 (V)	JQ783223	347S2 (V)	JQ783260	26S4 (II)	JQ783325
265S2 (V)	JQ783187	305S2 (V)	JQ783224	349S2 (V)	JQ783261	27S4 (I)	JQ783326
266S2 (V)	JQ783188	306S2 (V)	JQ783225	350S2 (V)	JQ783262	28S4 (I)	JQ783327
267S2 (V)	JQ783189	307S2 (V)	JQ783226	352S2 (V)	JQ783263	29S4 (II)	JQ783328
269S2 (V)	JQ783190	308S2 (V)	JQ783227	353S2 (V)	JQ783264	30S4 (II)	JQ783329
270S2 (V)	JQ783191	311S2 (V)	JQ783228	354S2 (V)	JQ783265	31S4 (I)	JQ783330
271S2 (V)	JQ783192	312S2 (V)	JQ783229	355S2 (V)	JQ783266	32S4 (II)	JQ783331
273S2 (V)	JQ783193	313S2 (V)	JQ783230	357S2 (V)	JQ783267	35S4 (I)	JQ783332
274S2 (V)	JQ783194	314S2 (V)	JQ783231	358S2 (V)	JQ783268	36S4 (II)	JQ783333

Table S5 GenBank nucleotide accession numbers for (a) selected soybean-nodulating bacteria from *A. bracteata* and *D. canadense* (*dna*K, *nod*C and *nif*H partial gene sequences) and (b) reference strains and selected isolates from soybean field sites A and B (*nif*H partial gene sequences).

Sequences in bold were generated in this study. For remainder of legend see Table S4.

(a) Isolates from native legumes				(b) Reference strains and isolates from soybeans (field sites A and B)	
Isolate No. (Lineage)	*dna*K	*nod*C	*nif*H	Isolate No. (Lineage / ST)	*nif*H
334S2 (V)	**JX013864**	**JX013895**	**JX013883**	HM155 (IV / ST 9)	**JX013875**
342S2 (V)	**JX013865**	**JX013896**	**JX013884**	HO172 (V / ST 25)	**JX013876**
355S2 (V)	**JX013866**	**JX013897**	**JX013885**	HO186 (I / ST 31)	**JX013877**
359S2 (V)	**JX013867**	**JX013898**	**JX013886**	HO196 (V / ST24)	**JX013878**
363S2 (V)	**JX013868**	**JX013899**	**JX013887**	HO199 (I / ST32)	**JX013879**
24S4 (II)	**JX013871**	**JX013902**	**JX013890**	OM28 (V / ST28)	**JX013880**
25S4 (II)	**-**	**JX013903**	**JX013891**	OM55 (II / ST21)	**JX013881**
27S4 (I)	**JX013872**	**JX013904**	**JX013892**	OO107 (II / ST15)	**JX013882**
28S4 (I)	**JX013873**	**JX013905**	**JX013893**	532C (V / ST 24)	**JX013874**
31S4 (I)	**-**	**JX013906**	**JX013894**	OO100 (III / ST34)	**JN186273**
				61A124	**JN186272**
				B. canariense LMG22265^T	EU818926
				B. cytisi LMG25866^T	GU001618
				B. elkanii USDA76^T	ab094963
				B. japonicum USDA6^T	NC017249
				B. japonicum USDA110	NC004463
				B. jicamae LMG24556^T	HM047127
				B. liaoningense LMG18230^T	EU818925
				B. pachyrhizi LMG24246^T	HM047124
				B. yuanmingense LMG21827^T	EU818927

Table S6 Primers and DNA amplification conditions.

All primers were used for PCR amplification as well as sequencing. PCR cycling conditions were modified in this study.

Primer	Direction	Sequence 5'-3'	PCR cycling conditions	Reference
*atp*D294F<br>*atp*D782R	Forward<br>Reverse	ATCGGCGAGCCGGTCGACGA<br>GCCGACACTTCMGAACCNGCCTG	5 min 95°C, 34x (45 sec 95°C, 30 sec 70°C, 40 sec 72°C), 7 min 72°C	Gaunt *et al.* 2001<br>Vinuesa *et al.* 2005
*gln*II12F<br>*gln*IITSr	Forward<br>Reverse	YAAGCTCGAGTACATYTGGCT<br>SGAGCCGTTCCAGTCGGTGTCG	5 min 95°C, 34x (45 sec 95°C, 30 sec 68°C, 45 sec 72°C), 7 min 72°C	Vinuesa *et al.* 2005<br>Stepkowski *et al.* 2005
*rec*A63F<br>*rec*A555R	Forward<br>Reverse	ATCGAGCGGTCGTTCGGCAAGGG<br>CGRATCTGGTTGATGAAGATCACCAT	5 min 95°C, 34x (45 sec 95°C, 30 sec 71°C, 40 sec 72°C), 7 min 72°C	Gaunt *et al.* 2001<br>Gaunt *et al.* 2001
*gyr*B343F<br>*gyr*B1043R	Forward<br>Reverse	TTCGACCAGAAYTCCTAYAAGG<br>AGCTTGTCCTTSGTCTGCG	5 min 95°C, 34x (45 sec 95°C, 30 sec 59°C, 50 sec 72°C), 7 min 72°C	Martens *et al.* 2008<br>Martens *et al.* 2008
*rpo*B454F<br>*rpo*B1364R	Forward<br>Reverse	ATCGTCTCGCAGATGCACCG<br>TCGATGTCGTCGATYTCGCC	5 min 95°C, 34x (45 sec 95°C, 30 sec 66°C, 1 min 72°C), 7 min 72°C	Vinuesa *et al.* 2008<br>Vinuesa *et al.* 2008
*dna*Kf<br>*dna*Kr	Forward<br>Reverse	TTCGACATCGACGCSAACGG<br>GCCTGCTGCKTGTACATGGC	5 min 95°C, 34x (45 sec 95°C, 30 sec 69°C, 30 sec 72°C), 7 min 72°C	Menna *et al.* 2009<br>Menna *et al.* 2009
*nod*CF4<br>*nod*CI	Forward<br>Reverse	AYGTHGGTYGAYGACGGATC<br>CGYGACAGCCANTCKCTATTG	5 min 95°C, 34x (45 sec 95°C, 30 sec 60°C, 1 min 72°C), 7 min 72°C	Islam *et al.* 2008<br>Islam *et al.* 2008
*nif*H40F<br>*nif*H817R	Forward<br>Reverse	GGNATCGGCAAGTCSACSAC<br>TCRAMCAGCATGTCCTCSAGCTC	5 min 95 °C, 34x (45 sec 95°C, 30 sec 69.2°C, 1 min 72°C), 7 min 72 °C	Vinuesa *et al.* 2005<br>Vinuesa *et al.* 2005

References for Table S6:

Gaunt, M. W., S. L. Turner, L. Rigottier-Gois, S. A. Lloyd-Macgilp, and E. Garay. 2001. Int. J. Syst. Evol. Microbiol. 51:2037–2048.

Islam, M. S., H. Kawasaki, Y. Muramatsu, Y. Nakagawa, and T. Seki. 2008. Biosci. Biotechnol. Biochem.72:1416–1429.

Martens, M., P. Dawyndt, R. Coopman, M. Gillis, P. De Vos, and A. Willems. 2008. Int. J. Syst. Evol. Microbiol. 58:200–214.

Menna, P., F. G. Barcellos, and M. Hungria. 2009. Int. J. Syst. Evol. Microbiol. 59:2934–2950.

Stepkowski, T., L. Moulin, A. Krzyzan´ ska, A. McInnes, I. J. Law, and J. Howieson. 2005. Appl. Environ. Microbiol. 71:7041–7052.

Vinuesa, P., C. Silva, D. Werner, and E. Martı´nez-Romero.2005. Mol. Phylogenet. Evol. 34:29–54.

Vinuesa, P., K. Rojas-Jime´nez, B. Contreras-Moreira, S. K. Mahna, B. N. Prasad, H. Moe et al. 2008. Appl. Environ. Microbiol. 74:6987–6996.

Table S7 Example of R scripts based on the phangorn phylogenetic package to test the extent of congruence between maximum-likelihood trees reconstructed for each of six housekeeping genes.

ML trees were reconstructed (GTR+G+I nucleotide substitution model) using 11 unique STs selected to represent the five STRUCTURE lineages in a tree inferred by ClonalFrame analysis (Fig 3a). For further description of method see Supplementary Fig S2.

```
library(phangorn)

###############################################################################
# First, we read in the data and trees for each data set.  With the given topology, we
# fit the model parameters for that data.
###############################################################################

# read data set and tree, and fit parameters for atpD

atpD11STs_data = read.phyDat("11STs_NoRef_atpD.fas.phylip", format = "sequential", type = "DNA")
atpD11STs_tree<-read.tree(file="11STs_NoRef_atpD.fas.phylip_phyml_tree_1000bs5rs.txt")
fit_atpD11STs = pml(atpD11STs_tree, data = atpD11STs_data)
fitGTR_atpD11STs = update(fit_atpD11STs, k=4, inv = 0.2)
fitGTR_atpD11STs = optim.pml(fitGTR_atpD11STs, FALSE, TRUE, TRUE, TRUE, TRUE, control = pml.control(trace = 0))

# read data set and tree, and fit parameters for dnaK

dnaK11STs_data = read.phyDat("11STs_NoRef_dnaK.fas.phylip", format = "sequential", type = "DNA")
dnaK11STs_tree<-read.tree(file="11STs_NoRef_dnaK.fas.phylip_phyml_tree_1000bs5rs.txt")
fit_dnaK11STs = pml(dnaK11STs_tree, data = dnaK11STs_data)
fitGTR_dnaK11STs = update(fit_dnaK11STs, k=4, inv = 0.2)
fitGTR_dnaK11STs = optim.pml(fitGTR_dnaK11STs, FALSE, TRUE, TRUE, TRUE, TRUE, control = pml.control(trace = 0))

# read data set and tree, and fit parameters for glnII

glnII11STs_data = read.phyDat("11STs_NoRef_glnII.fas.phylip", format = "sequential", type = "DNA")
glnII11STs_tree<-read.tree(file="11STs_NoRef_glnII.fas.phylip_phyml_tree_1000bs5rs.txt")
fit_glnII11STs = pml(glnII11STs_tree, data = glnII11STs_data)
fitGTR_glnII11STs = update(fit_glnII11STs, k=4, inv = 0.2)
fitGTR_glnII11STs = optim.pml(fitGTR_glnII11STs, FALSE, TRUE, TRUE, TRUE, TRUE, control = pml.control(trace = 0))

# read data set and tree, and fit parameters for gyrB

gyrB11STs_data = read.phyDat("11STs_NoRef_gyrB.fas.phylip", format = "sequential", type = "DNA")
gyrB11STs_tree<-read.tree(file="11STs_NoRef_gyrB.fas.phylip_phyml_tree_1000bs5rs.txt")
fit_gyrB11STs = pml(gyrB11STs_tree, data = gyrB11STs_data)
fitGTR_gyrB11STs = update(fit_gyrB11STs, k=4, inv = 0.2)
fitGTR_gyrB11STs = optim.pml(fitGTR_gyrB11STs, FALSE, TRUE, TRUE, TRUE, TRUE, control = pml.control(trace = 0))

# read data set and tree, and fit parameters for recA

recA11STs_data = read.phyDat("11STs_NoRef_recA.fas.phylip", format = "sequential", type = "DNA")
recA11STs_tree<-read.tree(file="11STs_NoRef_recA.fas.phylip_phyml_tree_1000bs5rs.txt")
fit_recA11STs = pml(recA11STs_tree, data = recA11STs_data)
fitGTR_recA11STs = update(fit_recA11STs, k=4, inv = 0.2)
fitGTR_recA11STs = optim.pml(fitGTR_recA11STs, FALSE, TRUE, TRUE, TRUE, TRUE, control = pml.control(trace = 0))

# read data set and tree, and fit parameters for rpoB

rpoB11STs_data = read.phyDat("11STs_NoRef_rpoB.fas.phylip", format = "sequential", type = "DNA")
rpoB11STs_tree<-read.tree(file="11STs_NoRef_rpoB.fas.phylip_phyml_tree_1000bs5rs.txt")
fit_rpoB11STs = pml(rpoB11STs_tree, data = rpoB11STs_data)
```

```
fitGTR_rpoB11STs = update(fit_rpoB11STs, k=4, inv = 0.2)
fitGTR_rpoB11STs = optim.pml(fitGTR_rpoB11STs, FALSE, TRUE, TRUE, TRUE, TRUE, control = pml.control(trace = 0))

##########################################################################
# Using the first data set, successively impose the trees inferred from other data sets,
# and fit model parameters.
##########################################################################

# first data set on second data set's tree
fit_atpD11STs_on_dnaK11STs_tree = pml(dnaK11STs_tree, data = atpD11STs_data)
fitGTR_atpD11STs_on_dnaK11STs_tree = update(fit_atpD11STs_on_dnaK11STs_tree, k=4, inv = 0.2)
fitGTR_atpD11STs_on_dnaK11STs_tree = optim.pml(fitGTR_atpD11STs_on_dnaK11STs_tree, FALSE, TRUE, TRUE, TRUE, TRUE,
control = pml.control(trace = 0))

# first data set on third data set's tree
fit_atpD11STs_on_glnII11STs_tree = pml(glnII11STs_tree, data = atpD11STs_data)
fitGTR_atpD11STs_on_glnII11STs_tree = update(fit_atpD11STs_on_glnII11STs_tree, k=4, inv = 0.2)
fitGTR_atpD11STs_on_glnII11STs_tree = optim.pml(fitGTR_atpD11STs_on_glnII11STs_tree, FALSE, TRUE, TRUE, TRUE, TRUE, control
= pml.control(trace = 0))

# first data set on forth data set's tree
fit_atpD11STs_on_gyrB11STs_tree = pml(gyrB11STs_tree, data = atpD11STs_data)
fitGTR_atpD11STs_on_gyrB11STs_tree = update(fit_atpD11STs_on_gyrB11STs_tree, k=4, inv = 0.2)
fitGTR_atpD11STs_on_gyrB11STs_tree = optim.pml(fitGTR_atpD11STs_on_gyrB11STs_tree, FALSE, TRUE, TRUE, TRUE, TRUE,
control = pml.control(trace = 0))

# first data set on fifth data set's tree
fit_atpD11STs_on_recA11STs_tree = pml(recA11STs_tree, data = atpD11STs_data)
fitGTR_atpD11STs_on_recA11STs_tree = update(fit_atpD11STs_on_recA11STs_tree, k=4, inv = 0.2)
fitGTR_atpD11STs_on_recA11STs_tree = optim.pml(fitGTR_atpD11STs_on_recA11STs_tree, FALSE, TRUE, TRUE, TRUE, TRUE, control
= pml.control(trace = 0))

# first data set on sixth (last) data set's tree
fit_atpD11STs_on_rpoB11STs_tree = pml(rpoB11STs_tree, data = atpD11STs_data)
fitGTR_atpD11STs_on_rpoB11STs_tree = update(fit_atpD11STs_on_rpoB11STs_tree, k=4, inv = 0.2)
fitGTR_atpD11STs_on_rpoB11STs_tree = optim.pml(fitGTR_atpD11STs_on_rpoB11STs_tree, FALSE, TRUE, TRUE, TRUE, TRUE,
control = pml.control(trace = 0))

##########################################################################
# Output difference of negative log-likelihood between the atpD tree/parameters, and the trees
# inferred from other data sets (but with remaining parameters re-fitted to the atpD data set).
##########################################################################

nll_atpD<--logLik(fitGTR_atpD11STs)
nll_atpD_on_dnaK<--logLik(fitGTR_atpD11STs_on_dnaK11STs_tree)
nll_atpD_on_glnII<--logLik(fitGTR_atpD11STs_on_glnII11STs_tree)
nll_atpD_on_gyrB<--logLik(fitGTR_atpD11STs_on_gyrB11STs_tree)
nll_atpD_on_recA<--logLik(fitGTR_atpD11STs_on_recA11STs_tree)
nll_atpD_on_rpoB<--logLik(fitGTR_atpD11STs_on_rpoB11STs_tree)
dnll<-(nll_atpD_on_dnaK - nll_atpD)
write.table(dnll, file="atpD11STs_vs_realOthers.dnll.txt", quote=FALSE, col.names=FALSE, row.names=FALSE)
dnll<-(nll_atpD_on_glnII - nll_atpD)
write.table(dnll, file="atpD11STs_vs_realOthers.dnll.txt", append=TRUE, quote=FALSE, col.names=FALSE, row.names=FALSE)
dnll<-(nll_atpD_on_gyrB - nll_atpD)
write.table(dnll, file="atpD11STs_vs_realOthers.dnll.txt", append=TRUE, quote=FALSE, col.names=FALSE, row.names=FALSE)
dnll<-(nll_atpD_on_recA - nll_atpD)
write.table(dnll, file="atpD11STs_vs_realOthers.dnll.txt", append=TRUE, quote=FALSE, col.names=FALSE, row.names=FALSE)
dnll<-(nll_atpD_on_rpoB - nll_atpD)
write.table(dnll, file="atpD11STs_vs_realOthers.dnll.txt", append=TRUE, quote=FALSE, col.names=FALSE, row.names=FALSE)

##########################################################################
# Using the second data set, successively impose the trees inferred from other data sets,
# and fit model parameters.
##########################################################################
```

```
# second data set on first data set's tree
fit_dnaK11STs_on_atpD11STs_tree = pml(atpD11STs_tree, data = dnaK11STs_data)
fitGTR_dnaK11STs_on_atpD11STs_tree = update(fit_dnaK11STs_on_atpD11STs_tree, k=4, inv = 0.2)
fitGTR_dnaK11STs_on_atpD11STs_tree = optim.pml(fitGTR_dnaK11STs_on_atpD11STs_tree, FALSE, TRUE, TRUE, TRUE, TRUE,
control = pml.control(trace = 0))

# second data set on third data set's tree
fit_dnaK11STs_on_glnII11STs_tree = pml(glnII11STs_tree, data = dnaK11STs_data)

fitGTR_dnaK11STs_on_glnII11STs_tree = update(fit_dnaK11STs_on_glnII11STs_tree, k=4, inv = 0.2)
fitGTR_dnaK11STs_on_glnII11STs_tree = optim.pml(fitGTR_dnaK11STs_on_glnII11STs_tree, FALSE, TRUE, TRUE, TRUE, TRUE,
control = pml.control(trace = 0))

# second data set on forth data set's tree
fit_dnaK11STs_on_gyrB11STs_tree = pml(gyrB11STs_tree, data = dnaK11STs_data)
fitGTR_dnaK11STs_on_gyrB11STs_tree = update(fit_dnaK11STs_on_gyrB11STs_tree, k=4, inv = 0.2)
fitGTR_dnaK11STs_on_gyrB11STs_tree = optim.pml(fitGTR_dnaK11STs_on_gyrB11STs_tree, FALSE, TRUE, TRUE, TRUE, TRUE,
control = pml.control(trace = 0))

# second data set on fifth data set's tree
fit_dnaK11STs_on_recA11STs_tree = pml(recA11STs_tree, data = dnaK11STs_data)
fitGTR_dnaK11STs_on_recA11STs_tree = update(fit_dnaK11STs_on_recA11STs_tree, k=4, inv = 0.2)
fitGTR_dnaK11STs_on_recA11STs_tree = optim.pml(fitGTR_dnaK11STs_on_recA11STs_tree, FALSE, TRUE, TRUE, TRUE, TRUE,
control = pml.control(trace = 0))

# second data set on sixth (last) data set's tree
fit_dnaK11STs_on_rpoB11STs_tree = pml(rpoB11STs_tree, data = dnaK11STs_data)
fitGTR_dnaK11STs_on_rpoB11STs_tree = update(fit_dnaK11STs_on_rpoB11STs_tree, k=4, inv = 0.2)
fitGTR_dnaK11STs_on_rpoB11STs_tree = optim.pml(fitGTR_dnaK11STs_on_rpoB11STs_tree, FALSE, TRUE, TRUE, TRUE, TRUE,
control = pml.control(trace = 0))

################################################################################
# Output difference of negative log-likelihood between the dnaK tree/parameters, and the trees
# inferred from other data sets (but with remaining parameters re-fitted to the dnaK data set).
################################################################################

nll_dnaK<--logLik(fitGTR_dnaK11STs)
nll_dnaK_on_atpD<--logLik(fitGTR_dnaK11STs_on_atpD11STs_tree)
nll_dnaK_on_glnII<--logLik(fitGTR_dnaK11STs_on_glnII11STs_tree)
nll_dnaK_on_gyrB<--logLik(fitGTR_dnaK11STs_on_gyrB11STs_tree)
nll_dnaK_on_recA<--logLik(fitGTR_dnaK11STs_on_recA11STs_tree)
nll_dnaK_on_rpoB<--logLik(fitGTR_dnaK11STs_on_rpoB11STs_tree)
dnll<-(nll_dnaK_on_atpD - nll_dnaK)
write.table(dnll, file="dnaK11STs_vs_realOthers.dnll.txt", quote=FALSE, col.names=FALSE, row.names=FALSE)
dnll<-(nll_dnaK_on_glnII - nll_dnaK)
write.table(dnll, file="dnaK11STs_vs_realOthers.dnll.txt", append=TRUE, quote=FALSE, col.names=FALSE, row.names=FALSE)
dnll<-(nll_dnaK_on_gyrB - nll_dnaK)
write.table(dnll, file="dnaK11STs_vs_realOthers.dnll.txt", append=TRUE, quote=FALSE, col.names=FALSE, row.names=FALSE)
dnll<-(nll_dnaK_on_recA - nll_dnaK)
write.table(dnll, file="dnaK11STs_vs_realOthers.dnll.txt", append=TRUE, quote=FALSE, col.names=FALSE, row.names=FALSE)
dnll<-(nll_dnaK_on_rpoB - nll_dnaK)
write.table(dnll, file="dnaK11STs_vs_realOthers.dnll.txt", append=TRUE, quote=FALSE, col.names=FALSE, row.names=FALSE)

################################################################################
# Using the third data set, successively impose the trees inferred from other data sets,
# and fit model parameters.
################################################################################

# third data set on first data set's tree
fit_glnII11STs_on_atpD11STs_tree = pml(atpD11STs_tree, data = glnII11STs_data)
fitGTR_glnII11STs_on_atpD11STs_tree = update(fit_glnII11STs_on_atpD11STs_tree, k=4, inv = 0.2)
fitGTR_glnII11STs_on_atpD11STs_tree = optim.pml(fitGTR_glnII11STs_on_atpD11STs_tree, FALSE, TRUE, TRUE, TRUE, TRUE, control
= pml.control(trace = 0))

# third data set on second data set's tree
```

```
fit_glnII11STs_on_dnaK11STs_tree = pml(dnaK11STs_tree, data = glnII11STs_data)
fitGTR_glnII11STs_on_dnaK11STs_tree = update(fit_glnII11STs_on_dnaK11STs_tree, k=4, inv = 0.2)
fitGTR_glnII11STs_on_dnaK11STs_tree = optim.pml(fitGTR_glnII11STs_on_dnaK11STs_tree, FALSE, TRUE, TRUE, TRUE, TRUE,
control = pml.control(trace = 0))

# third data set on forth data set's tree
fit_glnII11STs_on_gyrB11STs_tree = pml(gyrB11STs_tree, data = glnII11STs_data)
fitGTR_glnII11STs_on_gyrB11STs_tree = update(fit_glnII11STs_on_gyrB11STs_tree, k=4, inv = 0.2)
fitGTR_glnII11STs_on_gyrB11STs_tree = optim.pml(fitGTR_glnII11STs_on_gyrB11STs_tree, FALSE, TRUE, TRUE, TRUE, TRUE,
control = pml.control(trace = 0))

# third data set on fifth data set's tree
fit_glnII11STs_on_recA11STs_tree = pml(recA11STs_tree, data = glnII11STs_data)
fitGTR_glnII11STs_on_recA11STs_tree = update(fit_glnII11STs_on_recA11STs_tree, k=4, inv = 0.2)
fitGTR_glnII11STs_on_recA11STs_tree = optim.pml(fitGTR_glnII11STs_on_recA11STs_tree, FALSE, TRUE, TRUE, TRUE, TRUE, control
= pml.control(trace = 0))

# third data set on sixth (last) data set's tree
fit_glnII11STs_on_rpoB11STs_tree = pml(rpoB11STs_tree, data = glnII11STs_data)
fitGTR_glnII11STs_on_rpoB11STs_tree = update(fit_glnII11STs_on_rpoB11STs_tree, k=4, inv = 0.2)
fitGTR_glnII11STs_on_rpoB11STs_tree = optim.pml(fitGTR_glnII11STs_on_rpoB11STs_tree, FALSE, TRUE, TRUE, TRUE, TRUE,
control = pml.control(trace = 0))

##############################################################################
# Output difference of negative log-likelihood between the glnII tree/parameters, and the trees
# inferred from other data sets (but with remaining parameters re-fitted to the glnII data set).
##############################################################################

nll_glnII<--logLik(fitGTR_glnII11STs)
nll_glnII_on_atpD<--logLik(fitGTR_glnII11STs_on_atpD11STs_tree)
nll_glnII_on_dnaK<--logLik(fitGTR_glnII11STs_on_dnaK11STs_tree)
nll_glnII_on_gyrB<--logLik(fitGTR_glnII11STs_on_gyrB11STs_tree)
nll_glnII_on_recA<--logLik(fitGTR_glnII11STs_on_recA11STs_tree)
nll_glnII_on_rpoB<--logLik(fitGTR_glnII11STs_on_rpoB11STs_tree)
dnll<-(nll_glnII_on_atpD - nll_glnII)
write.table(dnll, file="glnII11STs_vs_realOthers.dnll.txt", quote=FALSE, col.names=FALSE, row.names=FALSE)
dnll<-(nll_glnII_on_dnaK - nll_glnII)
write.table(dnll, file="glnII11STs_vs_realOthers.dnll.txt", append=TRUE, quote=FALSE, col.names=FALSE, row.names=FALSE)
dnll<-(nll_glnII_on_gyrB - nll_glnII)
write.table(dnll, file="glnII11STs_vs_realOthers.dnll.txt", append=TRUE, quote=FALSE, col.names=FALSE, row.names=FALSE)
dnll<-(nll_glnII_on_recA - nll_glnII)
write.table(dnll, file="glnII11STs_vs_realOthers.dnll.txt", append=TRUE, quote=FALSE, col.names=FALSE, row.names=FALSE)
dnll<-(nll_glnII_on_rpoB - nll_glnII)
write.table(dnll, file="glnII11STs_vs_realOthers.dnll.txt", append=TRUE, quote=FALSE, col.names=FALSE, row.names=FALSE)

##############################################################################
# Using the forth data set, successively impose the trees inferred from other data sets,
# and fit model parameters.
##############################################################################

# forth data set on first data set's tree
fit_gyrB11STs_on_atpD11STs_tree = pml(atpD11STs_tree, data = gyrB11STs_data)
fitGTR_gyrB11STs_on_atpD11STs_tree = update(fit_gyrB11STs_on_atpD11STs_tree, k=4, inv = 0.2)
fitGTR_gyrB11STs_on_atpD11STs_tree = optim.pml(fitGTR_gyrB11STs_on_atpD11STs_tree, FALSE, TRUE, TRUE, TRUE, TRUE,
control = pml.control(trace = 0))

# forth data set on second data set's tree
fit_gyrB11STs_on_dnaK11STs_tree = pml(dnaK11STs_tree, data = gyrB11STs_data)
fitGTR_gyrB11STs_on_dnaK11STs_tree = update(fit_gyrB11STs_on_dnaK11STs_tree, k=4, inv = 0.2)
fitGTR_gyrB11STs_on_dnaK11STs_tree = optim.pml(fitGTR_gyrB11STs_on_dnaK11STs_tree, FALSE, TRUE, TRUE, TRUE, TRUE,
control = pml.control(trace = 0))

# forth data set on third data set's tree
fit_gyrB11STs_on_glnII11STs_tree = pml(glnII11STs_tree, data = gyrB11STs_data)
fitGTR_gyrB11STs_on_glnII11STs_tree = update(fit_gyrB11STs_on_glnII11STs_tree, k=4, inv = 0.2)
```

```
fitGTR_gyrB11STs_on_glnII11STs_tree = optim.pml(fitGTR_gyrB11STs_on_glnII11STs_tree, FALSE, TRUE, TRUE, TRUE, TRUE,
control = pml.control(trace = 0))

# forth data set on fifth data set's tree
fit_gyrB11STs_on_recA11STs_tree = pml(recA11STs_tree, data = gyrB11STs_data)
fitGTR_gyrB11STs_on_recA11STs_tree = update(fit_gyrB11STs_on_recA11STs_tree, k=4, inv = 0.2)
fitGTR_gyrB11STs_on_recA11STs_tree = optim.pml(fitGTR_gyrB11STs_on_recA11STs_tree, FALSE, TRUE, TRUE, TRUE, TRUE,
control = pml.control(trace = 0))

# forth data set on sixth (last) data set's tree
fit_gyrB11STs_on_rpoB11STs_tree = pml(rpoB11STs_tree, data = gyrB11STs_data)
fitGTR_gyrB11STs_on_rpoB11STs_tree = update(fit_gyrB11STs_on_rpoB11STs_tree, k=4, inv = 0.2)
fitGTR_gyrB11STs_on_rpoB11STs_tree = optim.pml(fitGTR_gyrB11STs_on_rpoB11STs_tree, FALSE, TRUE, TRUE, TRUE, TRUE,
control = pml.control(trace = 0))

#############################################################################
# Output difference of negative log-likelihood between the gyrB tree/parameters, and the trees
# inferred from other data sets (but with remaining parameters re-fitted to the gyrB data set).
#############################################################################

nll_gyrB<--logLik(fitGTR_gyrB11STs)
nll_gyrB_on_atpD<--logLik(fitGTR_gyrB11STs_on_atpD11STs_tree)
nll_gyrB_on_dnaK<--logLik(fitGTR_gyrB11STs_on_dnaK11STs_tree)
nll_gyrB_on_glnII<--logLik(fitGTR_gyrB11STs_on_glnII11STs_tree)
nll_gyrB_on_recA<--logLik(fitGTR_gyrB11STs_on_recA11STs_tree)
nll_gyrB_on_rpoB<--logLik(fitGTR_gyrB11STs_on_rpoB11STs_tree)
dnll<-(nll_gyrB_on_atpD - nll_gyrB)
write.table(dnll, file="gyrB11STs_vs_realOthers.dnll.txt", quote=FALSE, col.names=FALSE, row.names=FALSE)
dnll<-(nll_gyrB_on_dnaK - nll_gyrB)
write.table(dnll, file="gyrB11STs_vs_realOthers.dnll.txt", append=TRUE, quote=FALSE, col.names=FALSE, row.names=FALSE)
dnll<-(nll_gyrB_on_glnII - nll_gyrB)
write.table(dnll, file="gyrB11STs_vs_realOthers.dnll.txt", append=TRUE, quote=FALSE, col.names=FALSE, row.names=FALSE)
dnll<-(nll_gyrB_on_recA - nll_gyrB)
write.table(dnll, file="gyrB11STs_vs_realOthers.dnll.txt", append=TRUE, quote=FALSE, col.names=FALSE, row.names=FALSE)
dnll<-(nll_gyrB_on_rpoB - nll_gyrB)
write.table(dnll, file="gyrB11STs_vs_realOthers.dnll.txt", append=TRUE, quote=FALSE, col.names=FALSE, row.names=FALSE)

#############################################################################
# Using the fifth data set, successively impose the trees inferred from other data sets,
# and fit model parameters.
#############################################################################

# fifth data set on first data set's tree
fit_recA11STs_on_atpD11STs_tree = pml(atpD11STs_tree, data = recA11STs_data)
fitGTR_recA11STs_on_atpD11STs_tree = update(fit_recA11STs_on_atpD11STs_tree, k=4, inv = 0.2)
fitGTR_recA11STs_on_atpD11STs_tree = optim.pml(fitGTR_recA11STs_on_atpD11STs_tree, FALSE, TRUE, TRUE, TRUE, TRUE, control
= pml.control(trace = 0))

# fifth data set on second data set's tree
fit_recA11STs_on_dnaK11STs_tree = pml(dnaK11STs_tree, data = recA11STs_data)
fitGTR_recA11STs_on_dnaK11STs_tree = update(fit_recA11STs_on_dnaK11STs_tree, k=4, inv = 0.2)
fitGTR_recA11STs_on_dnaK11STs_tree = optim.pml(fitGTR_recA11STs_on_dnaK11STs_tree, FALSE, TRUE, TRUE, TRUE, TRUE,
control = pml.control(trace = 0))

# fifth data set on third data set's tree
fit_recA11STs_on_glnII11STs_tree = pml(glnII11STs_tree, data = recA11STs_data)
fitGTR_recA11STs_on_glnII11STs_tree = update(fit_recA11STs_on_glnII11STs_tree, k=4, inv = 0.2)
fitGTR_recA11STs_on_glnII11STs_tree = optim.pml(fitGTR_recA11STs_on_glnII11STs_tree, FALSE, TRUE, TRUE, TRUE, TRUE, control
= pml.control(trace = 0))

# fifth data set on forth data set's tree
fit_recA11STs_on_gyrB11STs_tree = pml(gyrB11STs_tree, data = recA11STs_data)
fitGTR_recA11STs_on_gyrB11STs_tree = update(fit_recA11STs_on_gyrB11STs_tree, k=4, inv = 0.2)
fitGTR_recA11STs_on_gyrB11STs_tree = optim.pml(fitGTR_recA11STs_on_gyrB11STs_tree, FALSE, TRUE, TRUE, TRUE, TRUE,
control = pml.control(trace = 0))
```

```
# fifth data set on sixth (last) data set's tree
fit_recA11STs_on_rpoB11STs_tree = pml(rpoB11STs_tree, data = recA11STs_data)
fitGTR_recA11STs_on_rpoB11STs_tree = update(fit_recA11STs_on_rpoB11STs_tree, k=4, inv = 0.2)
fitGTR_recA11STs_on_rpoB11STs_tree = optim.pml(fitGTR_recA11STs_on_rpoB11STs_tree, FALSE, TRUE, TRUE, TRUE, TRUE,
control = pml.control(trace = 0))

##########################################################################
# Output difference of negative log-likelihood between the recA tree/parameters, and the trees
# inferred from other data sets (but with remaining parameters re-fitted to the recA data set).
##########################################################################

nll_recA<--logLik(fitGTR_recA11STs)
nll_recA_on_atpD<--logLik(fitGTR_recA11STs_on_atpD11STs_tree)
nll_recA_on_dnaK<--logLik(fitGTR_recA11STs_on_dnaK11STs_tree)
nll_recA_on_glnII<--logLik(fitGTR_recA11STs_on_glnII11STs_tree)
nll_recA_on_gyrB<--logLik(fitGTR_recA11STs_on_gyrB11STs_tree)
nll_recA_on_rpoB<--logLik(fitGTR_recA11STs_on_rpoB11STs_tree)
dnll<-(nll_recA_on_atpD - nll_recA)
write.table(dnll, file="recA11STs_vs_realOthers.dnll.txt", quote=FALSE, col.names=FALSE, row.names=FALSE)
dnll<-(nll_recA_on_dnaK - nll_recA)
write.table(dnll, file="recA11STs_vs_realOthers.dnll.txt", append=TRUE, quote=FALSE, col.names=FALSE, row.names=FALSE)
dnll<-(nll_recA_on_glnII - nll_recA)
write.table(dnll, file="recA11STs_vs_realOthers.dnll.txt", append=TRUE, quote=FALSE, col.names=FALSE, row.names=FALSE)
dnll<-(nll_recA_on_gyrB - nll_recA)
write.table(dnll, file="recA11STs_vs_realOthers.dnll.txt", append=TRUE, quote=FALSE, col.names=FALSE, row.names=FALSE)
dnll<-(nll_recA_on_rpoB - nll_recA)
write.table(dnll, file="recA11STs_vs_realOthers.dnll.txt", append=TRUE, quote=FALSE, col.names=FALSE, row.names=FALSE)

##########################################################################
# Using the sixth data set, successively impose the trees inferred from other data sets,
# and fit model parameters.
##########################################################################

# sixth data set on second data set's tree
fit_rpoB11STs_on_atpD11STs_tree = pml(atpD11STs_tree, data = rpoB11STs_data)
fitGTR_rpoB11STs_on_atpD11STs_tree = update(fit_rpoB11STs_on_atpD11STs_tree, k=4, inv = 0.2)
fitGTR_rpoB11STs_on_atpD11STs_tree = optim.pml(fitGTR_rpoB11STs_on_atpD11STs_tree, FALSE, TRUE, TRUE, TRUE, TRUE,
control = pml.control(trace = 0))

# sixth data set on second data set's tree
fit_rpoB11STs_on_dnaK11STs_tree = pml(dnaK11STs_tree, data = rpoB11STs_data)
fitGTR_rpoB11STs_on_dnaK11STs_tree = update(fit_rpoB11STs_on_dnaK11STs_tree, k=4, inv = 0.2)
fitGTR_rpoB11STs_on_dnaK11STs_tree = optim.pml(fitGTR_rpoB11STs_on_dnaK11STs_tree, FALSE, TRUE, TRUE, TRUE, TRUE,
control = pml.control(trace = 0))

# sixth data set on third data set's tree
fit_rpoB11STs_on_glnII11STs_tree = pml(glnII11STs_tree, data = rpoB11STs_data)
fitGTR_rpoB11STs_on_glnII11STs_tree = update(fit_rpoB11STs_on_glnII11STs_tree, k=4, inv = 0.2)
fitGTR_rpoB11STs_on_glnII11STs_tree = optim.pml(fitGTR_rpoB11STs_on_glnII11STs_tree, FALSE, TRUE, TRUE, TRUE, TRUE,
control = pml.control(trace = 0))

# sixth data set on forth data set's tree
fit_rpoB11STs_on_gyrB11STs_tree = pml(gyrB11STs_tree, data = rpoB11STs_data)
fitGTR_rpoB11STs_on_gyrB11STs_tree = update(fit_rpoB11STs_on_gyrB11STs_tree, k=4, inv = 0.2)
fitGTR_rpoB11STs_on_gyrB11STs_tree = optim.pml(fitGTR_rpoB11STs_on_gyrB11STs_tree, FALSE, TRUE, TRUE, TRUE, TRUE,
control = pml.control(trace = 0))

# sixth data set on fifth data set's tree
fit_rpoB11STs_on_recA11STs_tree = pml(recA11STs_tree, data = rpoB11STs_data)
fitGTR_rpoB11STs_on_recA11STs_tree = update(fit_rpoB11STs_on_recA11STs_tree, k=4, inv = 0.2)
fitGTR_rpoB11STs_on_recA11STs_tree = optim.pml(fitGTR_rpoB11STs_on_recA11STs_tree, FALSE, TRUE, TRUE, TRUE, TRUE,
control = pml.control(trace = 0))

##########################################################################
```

```
# Output difference of negative log-likelihood between the recA tree/parameters, and the trees
# inferred from other data sets (but with remaining parameters re-fitted to the recA data set).
################################################################################

nll_rpoB<--logLik(fitGTR_rpoB11STs)
nll_rpoB_on_atpD<--logLik(fitGTR_rpoB11STs_on_atpD11STs_tree)
nll_rpoB_on_dnaK<--logLik(fitGTR_rpoB11STs_on_dnaK11STs_tree)
nll_rpoB_on_glnII<--logLik(fitGTR_rpoB11STs_on_glnII11STs_tree)
nll_rpoB_on_gyrB<--logLik(fitGTR_rpoB11STs_on_gyrB11STs_tree)
nll_rpoB_on_recA<--logLik(fitGTR_rpoB11STs_on_recA11STs_tree)
dnll<-(nll_rpoB_on_atpD - nll_rpoB)
write.table(dnll, file="rpoB11STs_vs_realOthers.dnll.txt", quote=FALSE, col.names=FALSE, row.names=FALSE)
dnll<-(nll_rpoB_on_dnaK - nll_rpoB)
write.table(dnll, file="rpoB11STs_vs_realOthers.dnll.txt", append=TRUE, quote=FALSE, col.names=FALSE, row.names=FALSE)
dnll<-(nll_rpoB_on_glnII - nll_rpoB)
write.table(dnll, file="rpoB11STs_vs_realOthers.dnll.txt", append=TRUE, quote=FALSE, col.names=FALSE, row.names=FALSE)
dnll<-(nll_rpoB_on_gyrB - nll_rpoB)
write.table(dnll, file="rpoB11STs_vs_realOthers.dnll.txt", append=TRUE, quote=FALSE, col.names=FALSE, row.names=FALSE)
dnll<-(nll_rpoB_on_recA - nll_rpoB)
write.table(dnll, file="rpoB11STs_vs_realOthers.dnll.txt", append=TRUE, quote=FALSE, col.names=FALSE, row.names=FALSE)

################################################################################
# Output the difference of negative log-likelihood between the each tree/parameters, and a set
# of random tree topologies (but with remaining parameters re-fitted to each data set)
################################################################################

for (i in 1:100)	{

	randTree<-rtree(11, rooted=FALSE, tip.label = atpD11STs_tree$tip.label)

	fit_atpD11STs_on_randTree = pml(randTree, data = atpD11STs_data)
	fitGTR_atpD11STs_on_randTree = update(fit_atpD11STs_on_randTree, k=4, inv = 0.2)
	fitGTR_atpD11STs_on_randTree = optim.pml(fitGTR_atpD11STs_on_randTree, FALSE, TRUE, TRUE, TRUE, TRUE, control = 
pml.control(trace = 0))
	nll_atpD_on_randTree<--logLik(fitGTR_atpD11STs_on_randTree)
	dnll<-(nll_atpD_on_randTree - nll_atpD)
	write.table(dnll, file="atpD11STs_vs_randomOthers.dnll.txt", append=TRUE, quote=FALSE, col.names=FALSE, 
row.names=FALSE)

	fit_dnaK11STs_on_randTree = pml(randTree, data = dnaK11STs_data)
	fitGTR_dnaK11STs_on_randTree = update(fit_dnaK11STs_on_randTree, k=4, inv = 0.2)
	fitGTR_dnaK11STs_on_randTree = optim.pml(fitGTR_dnaK11STs_on_randTree, FALSE, TRUE, TRUE, TRUE, TRUE, control = 
pml.control(trace = 0))
	nll_dnaK_on_randTree<--logLik(fitGTR_dnaK11STs_on_randTree)
	dnll<-(nll_dnaK_on_randTree - nll_dnaK)
	write.table(dnll, file="dnaK11STs_vs_randomOthers.dnll.txt", append=TRUE, quote=FALSE, col.names=FALSE, 
row.names=FALSE)

	fit_glnII11STs_on_randTree = pml(randTree, data = glnII11STs_data)
	fitGTR_glnII11STs_on_randTree = update(fit_glnII11STs_on_randTree, k=4, inv = 0.2)
	fitGTR_glnII11STs_on_randTree = optim.pml(fitGTR_glnII11STs_on_randTree, FALSE, TRUE, TRUE, TRUE, TRUE, control = 
pml.control(trace = 0))
	nll_glnII_on_randTree<--logLik(fitGTR_glnII11STs_on_randTree)
	dnll<-(nll_glnII_on_randTree - nll_glnII)
	write.table(dnll, file="glnII11STs_vs_randomOthers.dnll.txt", append=TRUE, quote=FALSE, col.names=FALSE, 
row.names=FALSE)

	fit_gyrB11STs_on_randTree = pml(randTree, data = gyrB11STs_data)
	fitGTR_gyrB11STs_on_randTree = update(fit_gyrB11STs_on_randTree, k=4, inv = 0.2)
	fitGTR_gyrB11STs_on_randTree = optim.pml(fitGTR_gyrB11STs_on_randTree, FALSE, TRUE, TRUE, TRUE, TRUE, control = 
pml.control(trace = 0))
	nll_gyrB_on_randTree<--logLik(fitGTR_gyrB11STs_on_randTree)
	dnll<-(nll_gyrB_on_randTree - nll_gyrB)
	write.table(dnll, file="gyrB11STs_vs_randomOthers.dnll.txt", append=TRUE, quote=FALSE, col.names=FALSE, 
row.names=FALSE)
```

```
	fit_recA11STs_on_randTree = pml(randTree, data = recA11STs_data)
	fitGTR_recA11STs_on_randTree = update(fit_recA11STs_on_randTree, k=4, inv = 0.2)
	fitGTR_recA11STs_on_randTree = optim.pml(fitGTR_recA11STs_on_randTree, FALSE, TRUE, TRUE, TRUE, TRUE, control = pml.control(trace = 0))
	nll_recA_on_randTree<--logLik(fitGTR_recA11STs_on_randTree)
	dnll<-(nll_recA_on_randTree - nll_recA)
	write.table(dnll, file="recA11STs_vs_randomOthers.dnll.txt", append=TRUE, quote=FALSE, col.names=FALSE, row.names=FALSE)

	fit_rpoB11STs_on_randTree = pml(randTree, data = rpoB11STs_data)
	fitGTR_rpoB11STs_on_randTree = update(fit_rpoB11STs_on_randTree, k=4, inv = 0.2)
	fitGTR_rpoB11STs_on_randTree = optim.pml(fitGTR_rpoB11STs_on_randTree, FALSE, TRUE, TRUE, TRUE, TRUE, control = pml.control(trace = 0))
	nll_rpoB_on_randTree<--logLik(fitGTR_rpoB11STs_on_randTree)
	dnll<-(nll_rpoB_on_randTree - nll_rpoB)
	write.table(dnll, file="rpoB11STs_vs_randomOthers.dnll.txt", append=TRUE, quote=FALSE, col.names=FALSE, row.names=FALSE)
}
```

Table S8 Statistics for five separate runs of STRUCTURE using the admixture model (independent allele frequencies) for values of K between 2 and 7.

Each run consisted of 100,000 burn-in and 200,000 sampling iterations. K, number of ancestral populations; α, frequency of admixture proportions; Pr (X/K), probability of observing the data given K.

K	Run	Pr (X/*K*)	Mean *ln* likelihood	Variance of *ln* likelihood	Mean α
K = 2	1	-19761.4	-19149.2	1224.5	0.0375
	2	-19742.1	-19149.4	1185.5	0.0378
	3	-19783.9	-19149.7	1268.5	0.0378
	4	-19782.2	-19149.3	1265.8	0.0376
	5	-19806.8	-19149.3	1315.1	0.0374
	Average	-19775.3	-19149.4	1251.9	0.0376
K = 3	1	-12938.8	-11932.3	2013.0	0.0262
	2	-12974.1	-11935.2	2078.0	0.0268
	3	-17621.2	-16730.4	1781.8	0.0248
	4	-17664.0	-16729.5	1869.0	0.0243
	5	-17661.5	-16730.4	1862.1	0.0247
	Average	-15771.9	-14811.6	1920.8	0.0254
K = 4	1	-10826.8	-9650.6	2352.3	0.0227
	2	-11119.4	-9816.1	2606.7	0.0251
	3	-10842.9	-9658.5	2368.7	0.0226
	4	-11195.6	-9816.2	2758.8	0.0250
	5	-11168.6	-9818.1	2701.0	0.0251
	Average	-11030.7	-9751.9	2557.5	0.0241
K = 5	1	-6885.5	-5440.1	2890.6	0.0220
	2	-7112.0	-5439.5	3345.1	0.0220
	3	-7080.1	-5439.7	3280.8	0.0222
	4	-7042.1	-5439.6	3205.0	0.0220
	5	-6885.0	-5438.0	2894.1	0.0219
	Average	-7000.9	-5439.4	3123.1	0.0220
K = 6	1	-7009.0	-5435.3	3147.4	0.0217
	2	-7204.3	-5435.7	3537.2	0.0216
	3	-7074.7	-5436.0	3277.3	0.0217
	4	-7082.9	-5435.0	3295.8	0.0216
	5	-7107.8	-5436.9	3341.7	0.0216
	Average	-7095.7	-5435.8	3319.9	0.0216
K = 7	1	-7370.8	-5595.9	3549.8	0.0217
	2	-7021.7	-5440.5	3162.5	0.0213
	3	-6951.1	-5438.8	3024.4	0.0214
	4	-7163.3	-5440.0	3446.6	0.0214
	5	-7085.9	-5439.4	3293.1	0.0214
	Average	-7118.6	-5470.9	3295.3	0.0214

Table S9 Genetic diversity statistics for *Bradyrhizobium* isolates from soybean cultivars M and O.

Hd, haplotype (gene) diversity. For explanation of other symbols see Table 3.

Soybean cultivar (No. of sequences)	Six concatenated core genes			Symbiotic *nod*C gene		
	h	Hd	π	h	Hd	π
Cultivar M (110)	17	0.842	0.0230	4	0.231	0.0098
Cultivar O (110)	28	0.921	0.0372	7	0.438	0.0274

Table S10 Genetic differentiation and gene flow statistics for selected core gene lineages based on six concatenated gene sequences.

Dxy, average number of nucleotide substitutions per site between lineages.
K_{ST}*, nucleotide sequence based statistic; P values based on permutation test (10,000 replications). F_{ST}, fixation index, and *Nm*, effective number of migrants.

Lineage comparison (no. of sequences)	No. of differences		*Dxy*	K_{ST}* (P value)	F_{ST}	*Nm*
	Fixed	Shared				
Lineage II *vs* IV (120, 53)	89	57	0.03947	0.51513 (0.0000)	0.92395	0.04
Lineage II *vs* V (120, 19)	18	36	0.02002	0.35729 (0.0000)	0.77337	0.15
Lineage IV *vs* V (53, 19)	63	36	0.03785	0.32965 (0.0000)	0.84741	0.09

Fig. S1 Maximum-likelihood tree of concatenated *atp*D-*gln*II-*rec*A-*gyr*B-*rpo*B-*dna*K partial gene sequences (3210 bp) for *Bradyrhizobium* reference strains and 35 unique STs representing soybean-nodulating bacteria from field sites A and B.

The tree was reconstructed using the GTR+G+I substitution model (1000 non-parametric bootstrap replications). Isolates are labelled according to ST number and field site of origin, respectively (*e.g.*18A, 21AB). STs and reference strains connected by under-scores possess the same multi-locus genotype; inoculant strains are shown in bold type. Roman numbers designate lineages inferred by STRUCTURE. Bootstrap values > 50% are shown at the nodes. Scale bar indicates estimated substitutions per site.

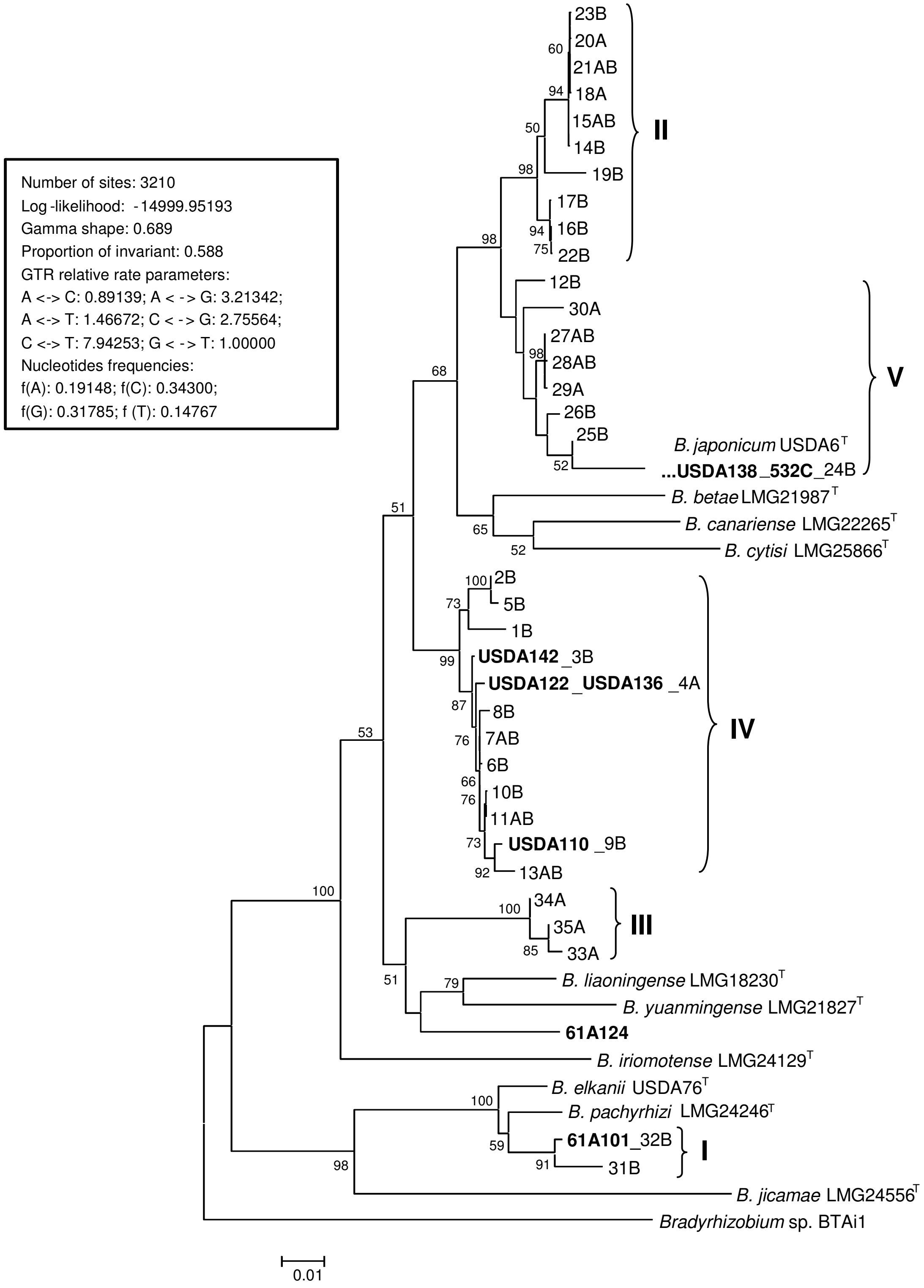

Fig. S2 Randomization test to assess congruence between maximum-likelihood (ML) trees of six housekeeping genes and 100 random trees.

The congruence test was carried out using datasets without (a) and with (b) reference strains consisting of *Bradyrhizobium* type and inoculant strains. Eleven unique STs (2, 4, 9, 11, 12, 15, 16, 25, 30, 31, 35) were selected to represent the different STRUCTURE lineages in the ClonalFrame phylogenetic tree (Fig 3a) and used to reconstruct ML trees for each of six housekeeping genes. The ML tree for each gene was compared with the ML trees of the other five genes. Shown are differences in log likelihood (Δ-*lnL*) between the ML tree for each gene and trees of the other five genes as well as between the tree of each gene and 100 random trees. Dotted lines indicate the 99th percentile of the likelihood differences between the ML tree of each gene and 100 random trees. For both datasets (a) and (b) only the topology of the *gyr*B gene tree is as different from trees of the other 5 genes as are random trees. Solid rectangles represent random trees and solid diamonds represent ML trees.

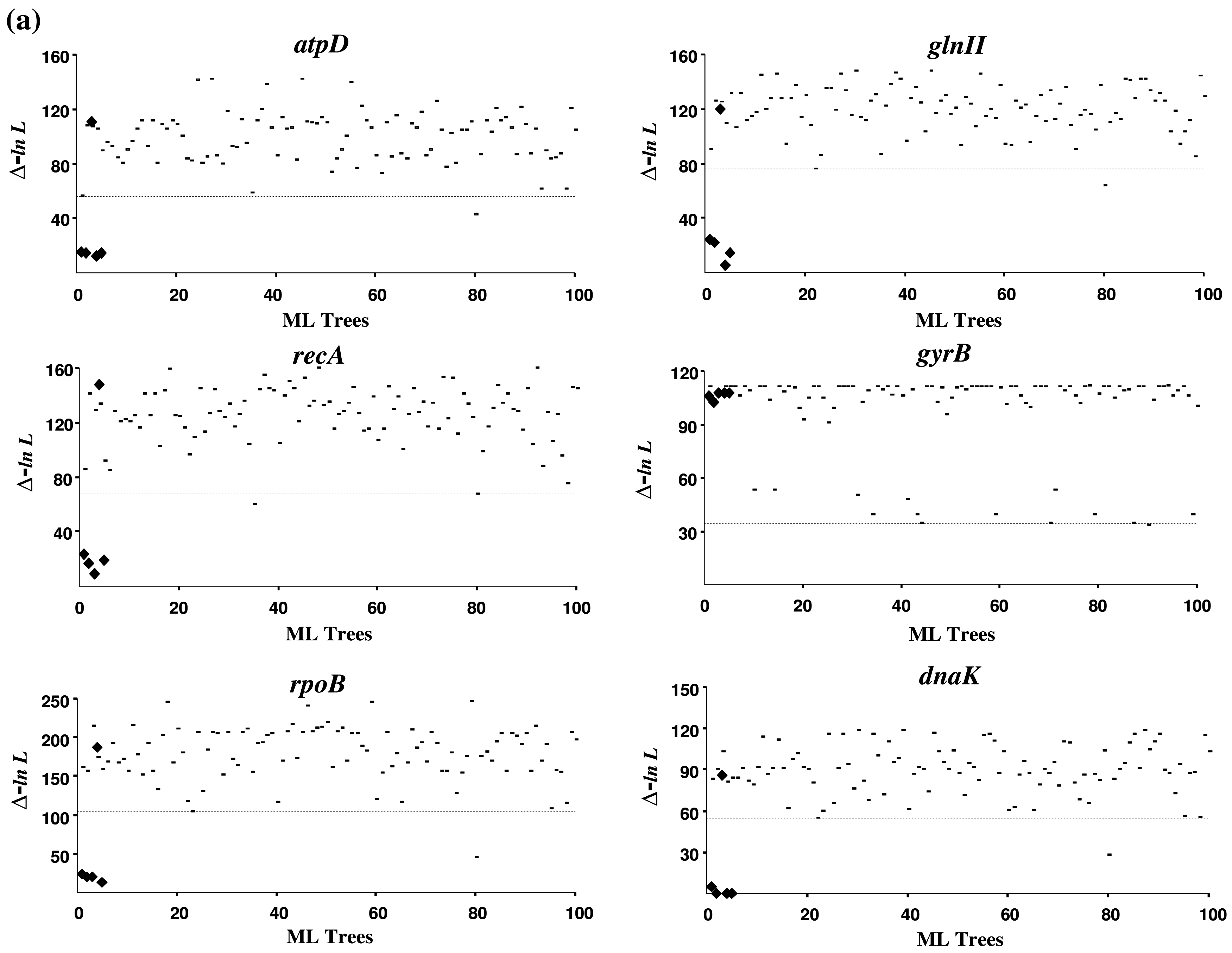

(a)
atpD
glnII
recA
gyrB
rpoB
dnaK
Δ-ln L
ML Trees
160
120
80
40
0
20
40
60
80
100
90
60
30
250
200
150
100
50
150

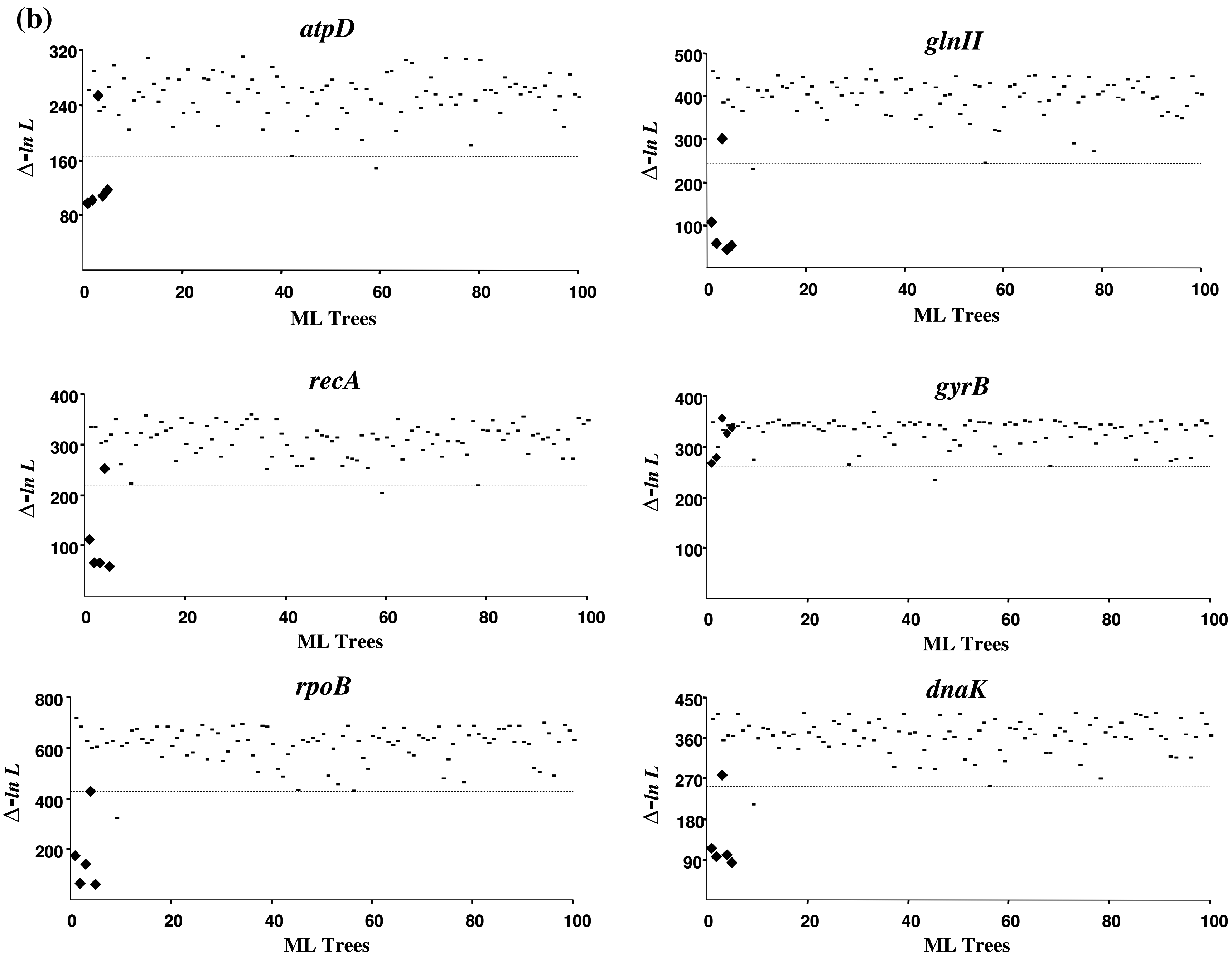
(b)
atpD
glnII
recA
gyrB
rpoB
dnaK
Δ-ln L
ML Trees
320
240
160
80
500
400
300
200
100
800
600
450
360
270
180
90
0
20
40
60
80
100

Fig. S3 Distribution of external to internal branch length ratios of a tree of lineage II (*n* = 120) inferred by ClonalFrame relative to that expected under coalescent simulation.

The external to internal branch length ratio (0.66, P = 0.007) is greater than that expected based on coalescent simulation indicating that the tree of lineage II is unexpectedly star-shaped, consistent with a recent clonal expansion or partial selective sweep (see Fig. 4).

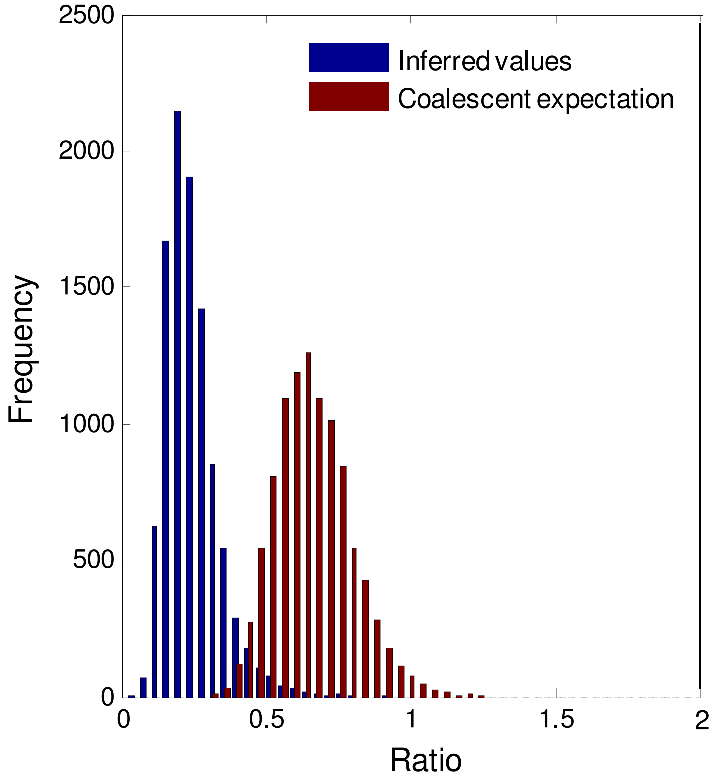

Fig. S4 Maximum-likelihood tree of unique *gyr*B partial gene sequences (618 bp) of *Bradyrhizobium* isolates from soybean field sites A and B and reference taxa showing putative xenologous sequences.

Isolates are labelled according to ST number and those connected by underscores have the same sequence. STs shown in color are incongruent with the "species" tree topology of six concatenated housekeeping gene sequences (Fig S1). Ten putative xenologous sequences (shown in red) are highly supported (bootstrap values > 95%). Roman numbers in parentheses designate lineages inferred by STRUCTURE. Type strains (designated superscript T) and inoculant strains are indicated. Values > 50% (1000 non-parametric bootstrap replications) are shown at nodes. For remainder of legend see Fig S1

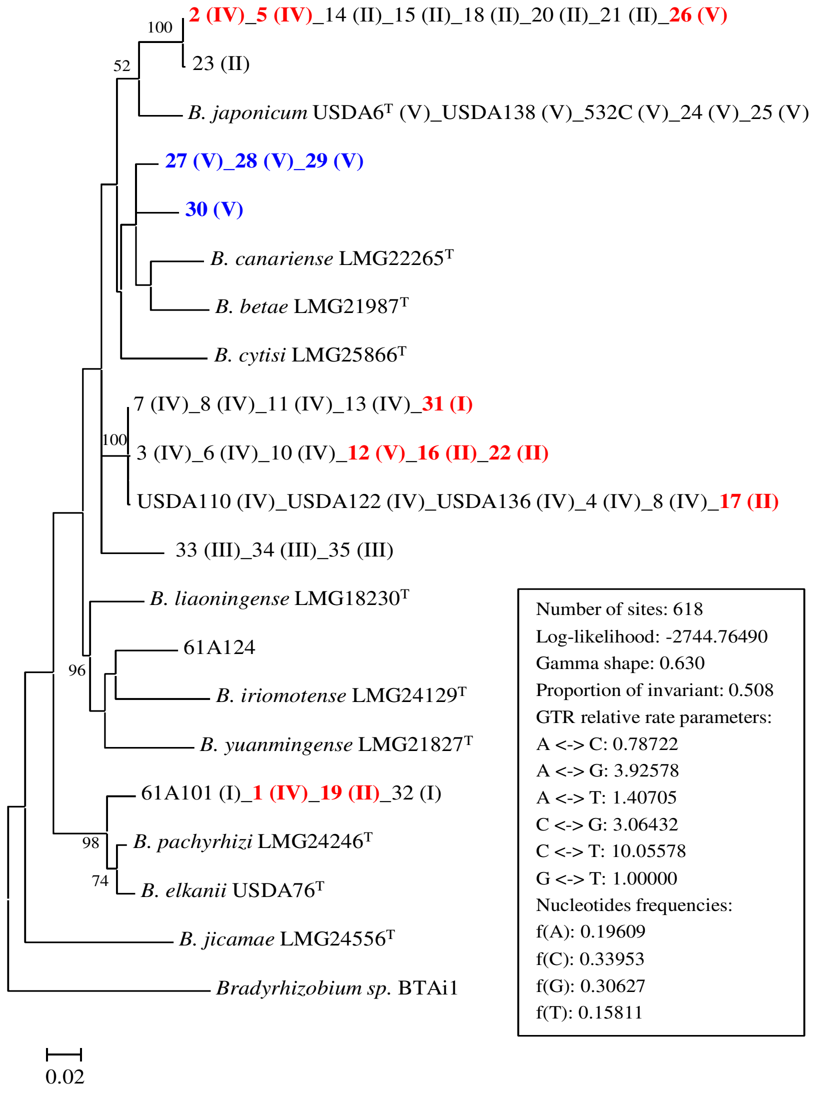

Fig. S5 Maximum-likelihood phylogenetic trees of (a) *rec*A (462 bp) and (b) concatenated *rec*A-*dna*K (831 bp) partial gene sequences representing soybean-nodulating bacteria from *A. bracteata* and *D. canadense*, from soybean field sites A and B, and, reference taxa.

Tree (a): *rec*A sequences of 148 bacterial isolates from native legumes were analyzed. Sequences of selected isolates from *D canadense* and *A. bracteata* are shown in blue and red, respectively; values in parentheses represent the number of sequences analyzed (See Table S4).
Tree (b): concatenated sequences of selected isolates from native legumes were analyzed (see Table S5) and are color coded as for tree (a).
Roman numbers represent lineages inferred by STRUCTURE. Isolates from soybeans at field sites A and B are labelled according to ST number. Sequences connected by a forward slash symbol are identical. Type strains (designated superscript T) and inoculant strains are indicated. Values > 50% (1000 non-parametric bootstrap replications) are shown at nodes. For remainder of legend see Fig S1.

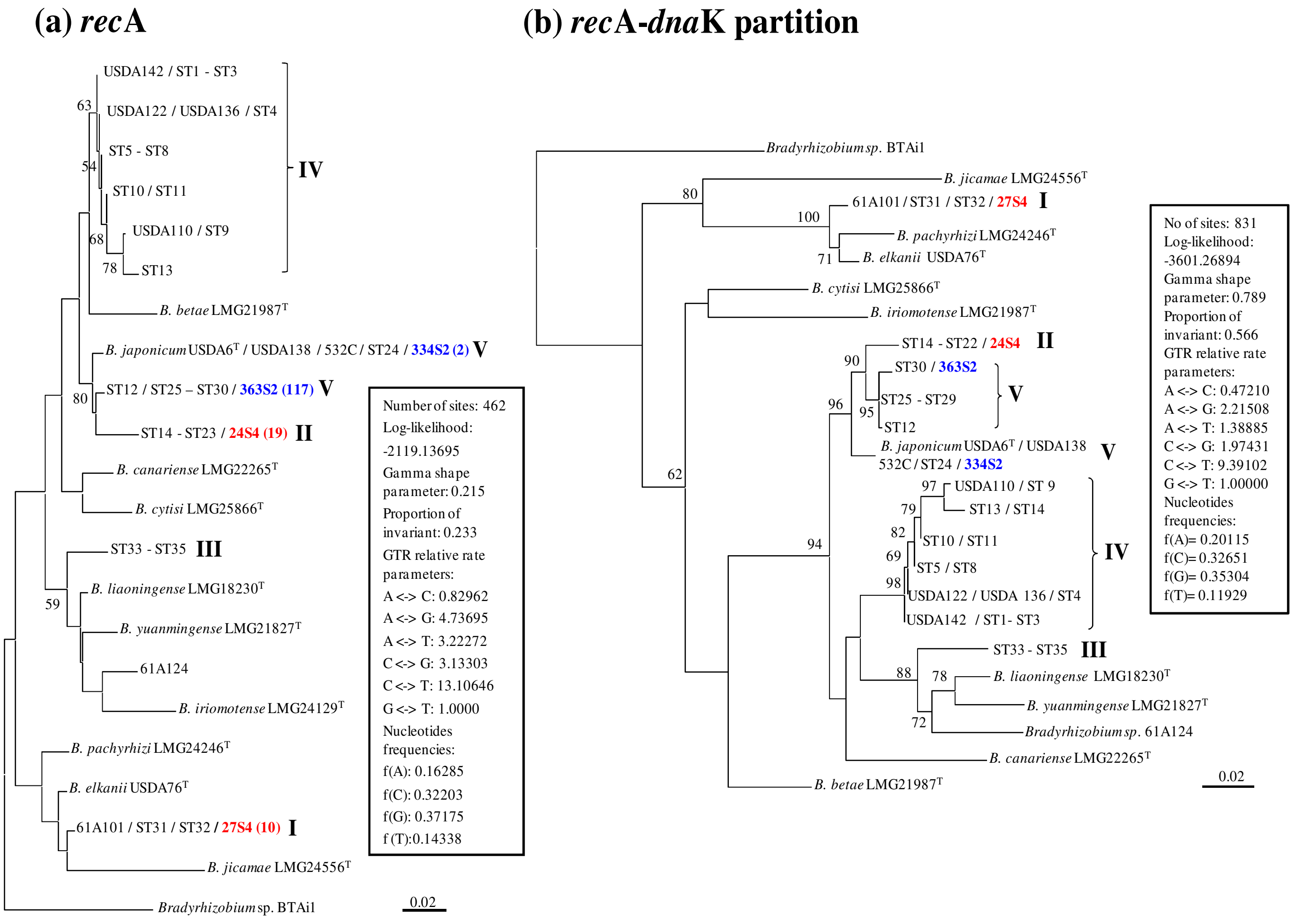

Supplement: Supplementary file 1 [file ece30002-2943-SD1.pdf]
