# Supplementary figures and images for: Microevolution of symbiotic Bradyrhizobium populations associated with soybeans in east North America
Source: Ecol Evol. 2012 Oct 22;2(12):2943–61. doi: 10.1002/ece3.404 (PMC3538991; doi:10.1002/ece3.404)

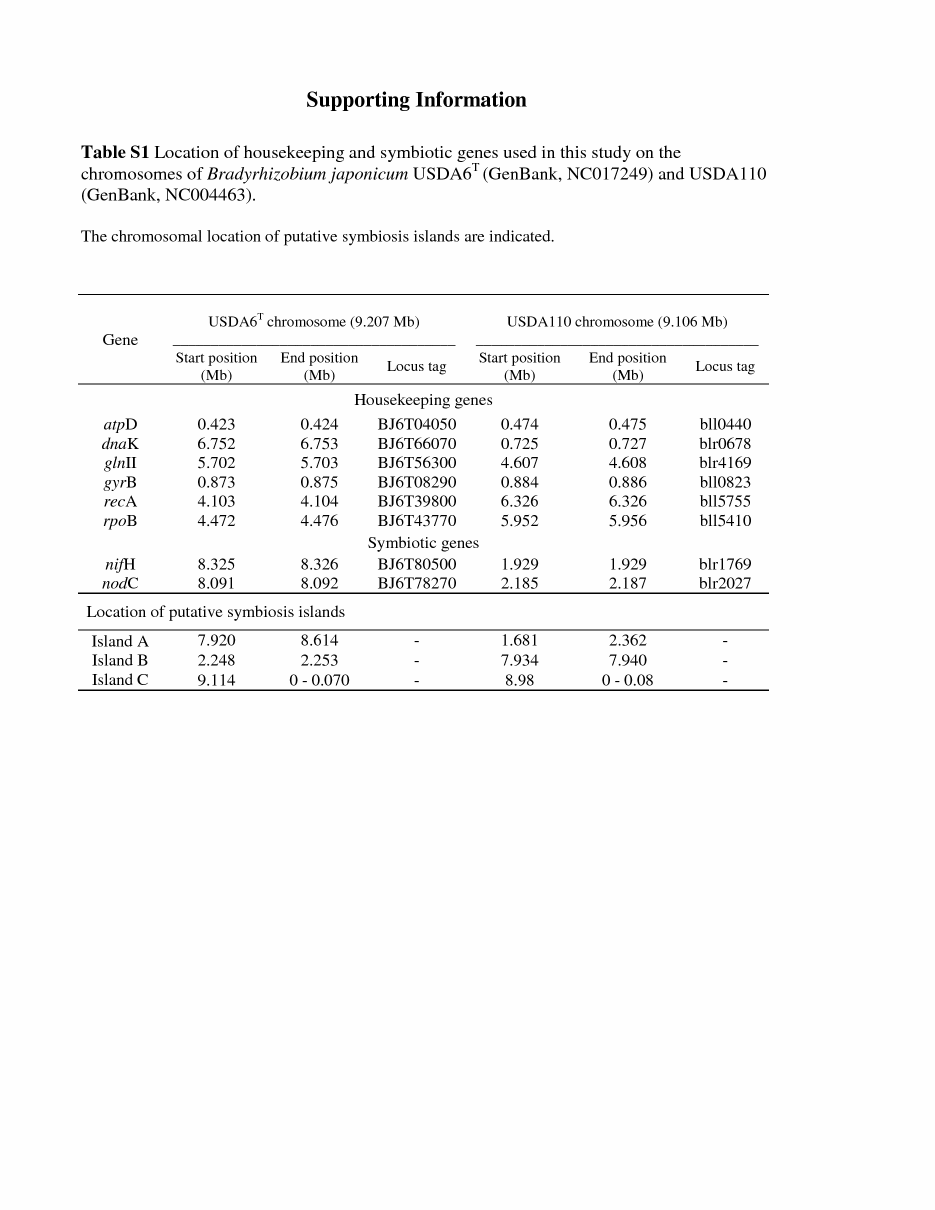

Supplement: Supplementary file 2 [file ece30002-2943-SD2.png]
